# Lentivirus-mediated gene therapy corrects ribosomal biogenesis and shows promise for Diamond Blackfan anemia

**DOI:** 10.1172/jci.insight.171650

**Published:** 2024-05-22

**Authors:** Yari Giménez, Manuel Palacios, Rebeca Sánchez-Domínguez, Christiane Zorbas, Jorge Peral, Alexander Puzik, Laura Ugalde, Omaira Alberquilla, Mariela Villanueva, Paula Río, Eva Gálvez, Lydie Da Costa, Marion Strullu, Albert Catala, Anna Ruiz-Llobet, Jose Carlos Segovia, Julián Sevilla, Brigitte Strahm, Charlotte M. Niemeyer, Cristina Beléndez, Thierry Leblanc, Denis L.J. Lafontaine, Juan Bueren, Susana Navarro

**Affiliations:** 1 Division of Hematopoietic Innovative Therapies, CIEMAT, Madrid, Spain.; 2 Instituto Nacional de Investigación Biomédica en Enfermedades Raras (CIBERER), Instituto de Salud Carlos III, Madrid, Spain.; 3 Advanced Therapies Unit, IIS-Fundación Jimenez Diaz (IIS-FJD, UAM), Madrid, Spain.; 4 RNA Molecular Biology, Fonds de la Recherche Scientifique (FRS/FNRS), Université libre de Bruxelles (ULB), Biopark campus, Gosselies, Belgium.; 5 Division of Pediatric Hematology and Oncology, Department of Pediatrics and Adolescent Medicine Medical Center, Faculty of Medicine, University of Freiburg, Freiburg, Germany.; 6 Hospital del Niño Jesús, Madrid, Spain.; 7 AP-HP, Hematology diagnostic laboratory, Hôpital Robert-Debré, Paris, France.; 8 University of Paris; Hematim, UR4666, UPJV; LABEX GR-EX, Paris, France.; 9 AP-HP, service Immuno-Hématologie pédiatique, Hôpital R. Debré, Paris, France.; 10 Hospital San Joan D′Deu, Barcelona, Spain.; 11 Sección de Hematología y Oncología Pediátricas, Hospital General Universitario Gregorio Marañón, Madrid, Spain.; 12 Facultad de Medicina, Universidad Complutense de Madrid, Madrid, Spain.; 13 Instituto Investigación Sanitaria Gregorio Marañón, Madrid, Spain.

**Keywords:** Hematology, Therapeutics, Gene therapy, Genetic diseases, Hematopoietic stem cells

## Abstract

This study lays the groundwork for future lentivirus-mediated gene therapy in patients with Diamond Blackfan anemia (DBA) caused by mutations in ribosomal protein S19 (*RPS19*), showing evidence of a new safe and effective therapy. The data show that, unlike patients with Fanconi anemia (FA), the hematopoietic stem cell (HSC) reservoir of patients with DBA was not significantly reduced, suggesting that collection of these cells should not constitute a remarkable restriction for DBA gene therapy. Subsequently, 2 clinically applicable lentiviral vectors were developed. In the former lentiviral vector, *PGK.CoRPS19 LV*, a codon-optimized version of *RPS19* was driven by the phosphoglycerate kinase promoter (*PGK*) already used in different gene therapy trials, including FA gene therapy. In the latter one, *EF1α.CoRPS19 LV*, RPS19 expression was driven by the elongation factor alpha short promoter, *EF1*α*(s)*. Preclinical experiments showed that transduction of DBA patient CD34^+^ cells with the *PGK.CoRPS19 LV* restored erythroid differentiation, and demonstrated the long-term repopulating properties of corrected DBA CD34^+^ cells, providing evidence of improved erythroid maturation. Concomitantly, long-term restoration of ribosomal biogenesis was verified using a potentially novel method applicable to patients’ blood cells, based on ribosomal RNA methylation analyses. Finally, in vivo safety studies and proviral insertion site analyses showed that lentivirus-mediated gene therapy was nontoxic.

## Abstract 2

Clinically applicable lentiviral vectors restore characteristic defects of hematopoietic stem cells from RPS19-deficient Diamond-Blackfan anemia patients.

## Graphical abstract

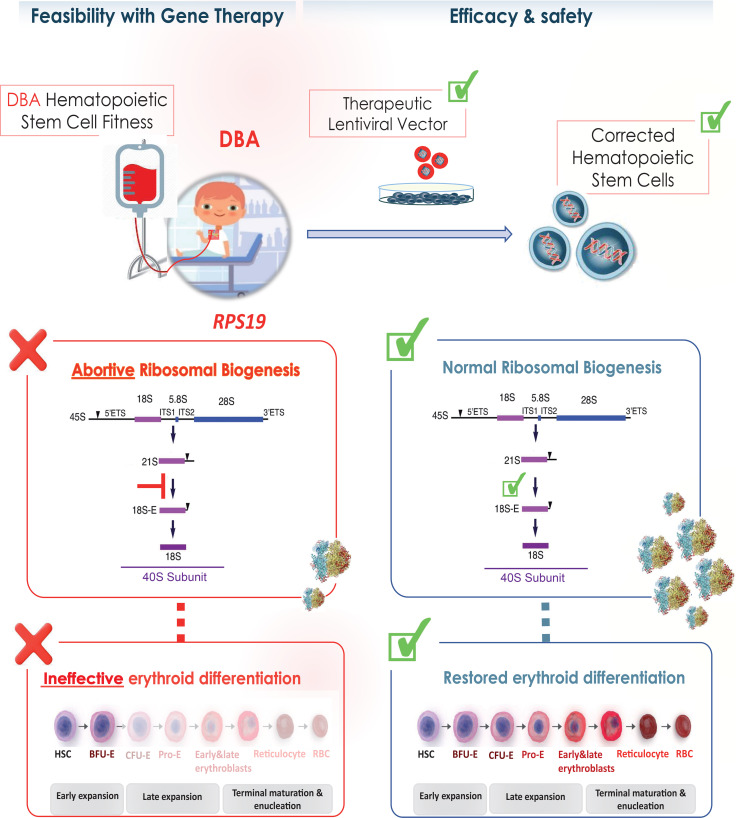

## Introduction

Diamond Blackfan anemia (DBA, OMIM #105650) is a bone marrow failure (BMF) syndrome primarily characterized by erythroblastopenia (1). Additionally, an increased incidence of myelodysplastic syndrome, acute myeloid leukemia, and solid tumors has been reported in patients with DBA (2). The estimated prevalence of DBA is 7 cases per million live births (1). Most cases are associated with mutations in any of the 6 ribosomal protein (RP) genes (*RPS19*, *RPL5*, *RPS26*, *RPL11*, *RPL35A*, and *RPS24*). In fact, mutations in any of the 24 of the 80 genes encoding RPs, and in any of the 11 genes encoding for the small ribosomal subunits or in the 13 genes encoding for the large subunits, have been implicated in DBA (3). The most frequently mutated RP gene is *RPS19* (25% of all DBA cases) (1). Additionally, recent reports have described DBA-causing mutations in factors involved in RP transport and assembly (e.g., *TSR2* and *HEATR3*) (4–10) and in regulators of these functions (certain allelic variants of *GATA1*, *EPO*, and *p53* are associated with a DBA-like phenotype).

Allogeneic hematopoietic stem cell transplantation (HSCT) is currently the only curative option for correcting the hematological signs of the disease, either in patients who are nonresponsive to steroids or in cases of clonal evolution. However, this treatment is mainly applicable to pediatric patients younger than the age of 10 (11). Previous clinical studies have shown that gene therapy (GT) based on transduction of autologous hematopoietic stem cells (HSCs) with therapeutic lentiviral vectors (LVs) constitutes a good alternative to HSCT in different monogenic disorders (12). In the field of BMF syndromes, only in Fanconi anemia (FA) have clinical GT programs shown preliminary evidence of efficacy and safety (13). In that case, gene-complemented CD34^+^ cells were infused in the patients in the absence of any conditioning regimen. The specific design of a nonconditioning GT approach was proposed for FA because of the proliferative advantage of corrected HSCs observed in preclinical experiments in which transduced FA HSCs were infused into immunodeficient mice (14). In recent FA GT trials, most patients showed progressive engraftment of corrected HSCs, facilitating stabilization and even correction of the BMF in the patients with the highest levels of engraftment (13, 15). Although the scarcity and poor repopulation ability of uncorrected HSCs from patients with FA limited the collection of clinically relevant numbers of HSCs for GT purposes in several patients (16), the severe HSC defects characteristic of FA constitute the basis of the HSC proliferative advantage associated with somatic mosaicism in FA (17–20) and with the efficacy of LV-mediated GT in the absence of conditioning (13).

As observed in patients with FA treated by GT, corrected DBA mouse HSCs by LVs showed a marked proliferative advantage (21–23). Nevertheless, whether the same applies to corrected HSCs from patients with DBA is unknown, since neither the mouse model nor healthy donor (HD) cells with impaired *RPS19* expression (23, 24) actually mimic the defects of human DBA patient HSCs. In addition, although some of the previous studies have used cells from patients with DBA, in these cases cells were transduced with not clinically applicable vectors since these used viral long terminal repeat directed (*LTR*-directed) viral promoters associated with genotoxicity risks (25, 26).

The aim of the present study was to conduct comprehensive GT experiments aiming at facilitating the initiation of GT trials in patients with DBA. As previously done in both preclinical (14) and clinical (13) FA GT studies, we have investigated systematically the phenotypic properties of HSPCs from patients with DBA and compared them both with HDs and with patients with FA. We have further performed in vitro and in vivo experiments in order to evaluate the efficacy and safety of LV-mediated GT in DBA. Our studies offer compelling evidence in support of GT as a novel therapeutic option for restoring the hematopoietic function of patients with DBA.

## Results

### Analysis of the HSC reservoir of bone marrow from patients with DBA.

First, peripheral blood cell (PBC) counts from patients with DBA with different RP gene mutations were evaluated and compared with PBC counts from HDs. Consistent with previous studies (1, 27), in patients with DBA, major hematological defects were found in the erythrocyte lineage, though significantly lower numbers of platelets and white blood cells also were observed (Supplemental Figure 1; supplemental material available online with this article; https://doi.org/10.1172/jci.insight.171650DS1).

**Figure 1:**
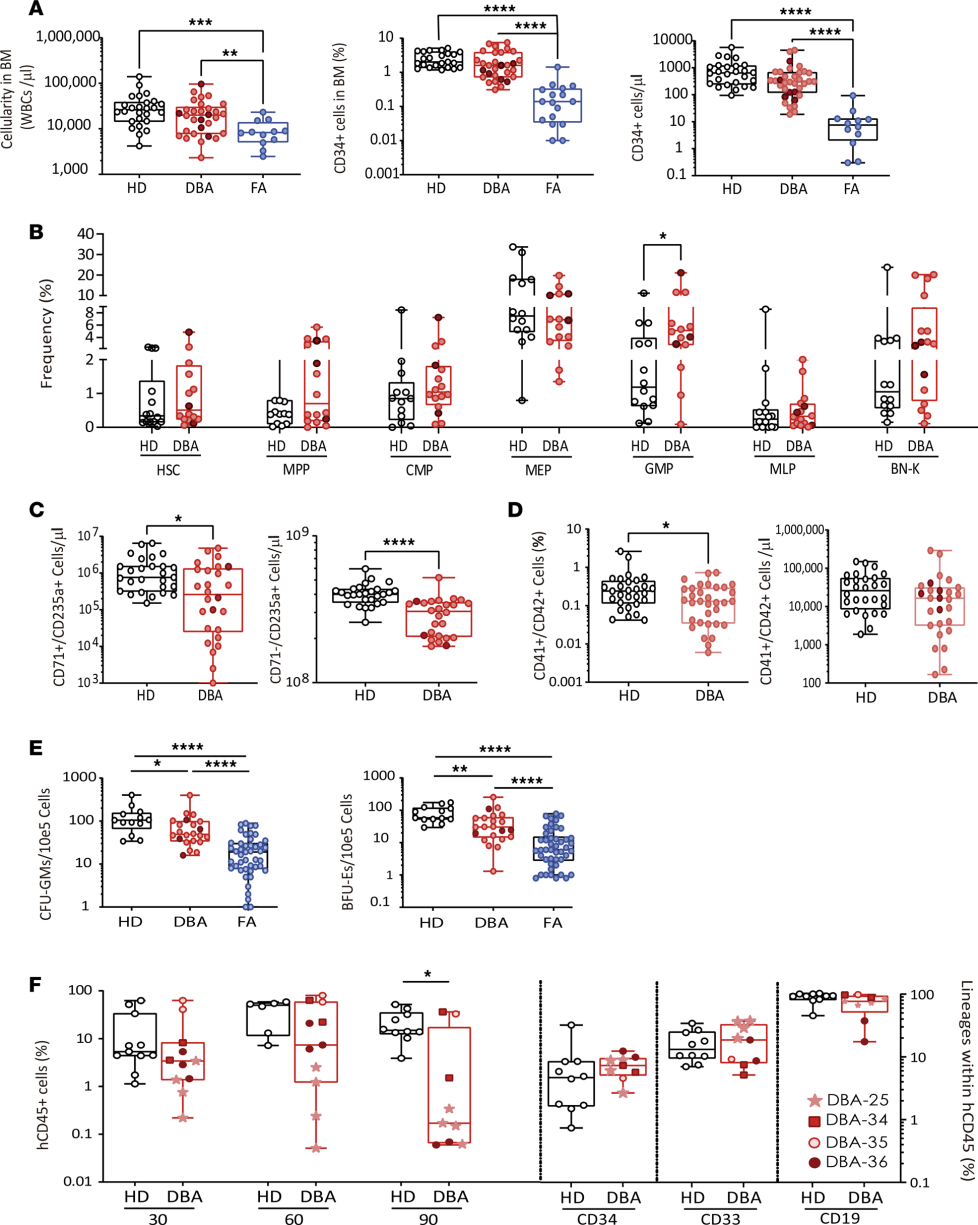
Analysis of the HSPC content and composition of bone marrow samples from patients with DBA. (**A**) Cellularity (WBCs/µL), CD34^+^ cell percentage, and CD34^+^ cell concentration (cells/µL) in bone marrow (BM) from HDs, patients with FA, and patients with DBA. (**B**) Frequency of hematopoietic stem cells (HSC, Lin^–^CD34^+^CD38^–^CD90^+^CD45RA^–^), multipotent progenitors (MPP, CD34^+^CD38^–^Thy-1^–^CD45RA^–^Flt3^+^CD7^–^CD10^–^), intermediate hematopoietic progenitors such as common myeloid progenitors (CMP, CD34^+^CD38^+^Thy-1^–^CD45RA^–^Flt3^+^CD7^–^CD10^–^), megakaryocytic and erythroid progenitors (MEP, CD34^+^CD38^+^Thy-1^–^CD45RA^–^Flt3^–^CD7^–^CD10^–^), granulocyte–monocyte progenitors (GMP, CD34^+^CD38^+^Thy-1^–^CD45RA^+^Flt3^+^CD7^–^CD10^–^), multilymphoid progenitors (MLP, CD34^+^CD38^–^Thy-1^lo^CD45RA^–^Flt3^+^CD7^–/+^CD10^–^), and B-NKs (CD34^+^CD38^–^Thy-1^lo^CD45RA^–^Flt3^+^CD7^–/+^CD10^+^). (**C**) CD71^+^CD235a^+^ and CD71^–^CD235a^+^ proerythroblast content (cells/μL) of BM from DBA patients compared with HDs. (**D**) CD41^+^CD42^+^ frequency and content of BM from patients with DBA compared with HDs. (**E**) Number of granulocyte-macrophage progenitor colony-forming units (CFU-GMs) per 10^5^ seeded mononuclear cells (MNCs) and number of burst-forming unit-erythroid progenitors (BFU-Es) per 10^5^ seeded MNCs. (**F**) Left: Percentage of human CD45^+^ (hCD45^+^) cells found in mouse BM at 3 time points posttransplantation (30, 60, and 90 days). Right: differentiation to the different lineages (CD33^+^: myeloid, CD19^+^: lymphoid, and CD34^+^: HSCs). The graphs show the median and interquartile range along with the 90th and 10th percentiles. In both graphs, each symbol represents 1 patient. The degree of statistical significance was determined by multiple-comparison Kruskal-Wallis test (*P* value; * ≤ 0.017; ** ≤ 0.003; *** ≤ 0.0003; **** ≤ 0.00003) for figures **A** and **E**. The degree of significance was determined with the 2-tailed Mann-Whitney test (*P* value; * ≤ 0.05; ** ≤ 0.01; *** ≤ 0.001; **** ≤ 0.0001) for **B**–**D**. Patients with DBA marked with darker dots were under corticosteroid treatment.

To characterize the HSC compartment of these patients, we studied the proportion and number of CD34^+^ cells in their bone marrow (BM) and compared with values obtained in HDs and patients with FA. In marked contrast with findings in patients with FA, who displayed very low numbers of CD34^+^ cells, the proportion and total number of CD34^+^ progenitors in patients with DBA were very similar to those determined in HDs (Figure 1A). Further analyses to compare the content of HSPCs in BM samples from patients with DBA and HDs were performed as previously described (16). They could not be done on FA samples because of their insufficient cellularity and CD34^+^ cell content. Our analyses showed that BM samples from HDs and patients with DBA had a similar proportion of multipotent progenitors (MPPs) and of other intermediate precursors such as the common myeloid (CMPs), multilymphoid (MLPs), and B lymphoid and NK progenitors (B-NKs) (Figure 1B). A slight decrease in erythro-megakaryocytic progenitors (MEPs), concomitant with a significant increase in the proportion of granulocyte-monocyte progenitors (GMPs), was noted in DBA versus HD BM samples. Additionally, we saw significant decreases in CD71^+^CD235a^+^ and CD71^–^CD235a^+^ erythroid progenitors (Figure 1C) and in CD41^–^CD42^+^ megakaryocytes (Figure 1D) in patients with DBA versus HDs.

Clonogenic assays showed that BM from patients with DBA contained a lower proportion of myeloid progenitor cells (fewer granulocyte-macrophage colony-forming units, CFU-GMs) than HDs and a more marked reduction in the content of erythroid progenitors (BFU-E). In both cases the comparison with patients with FA showed that numbers of these progenitor cells were markedly lower for patients with FA than for DBA (Figure 1E).

To evaluate the functional properties of primitive DBA HSCs, we compared the in vivo repopulating ability of BM CD34^+^ cells from HDs and patients with DBA in immunodeficient *Cg-Prkdcscid Il2rgtm1Wjl/SzJ* (NSG) mice (DBA-25, DBA-34, DBA-35, and DBA-36). As shown in Figure 1F, similar levels of human CD45^+^ cells were observed in mice that had been transplanted with samples from HDs and patients with DBA, except at day 90 postinfusion, which showed lower levels of human CD45^+^ cells in recipients of DBA samples. Despite this difference, comparable proportions of human CD34^+^ cells and B cells were found in both recipient groups. These results suggest that the repopulating ability of uncorrected HSCs from patients with DBA might be reduced as compared with HD HSCs in the long term. Nevertheless, the observed defects were markedly less severe than those previously determined in FA HSCs, which were only capable of repopulating NSG mouse hematopoiesis after gene correction (14).

### Restored ribosomal biogenesis in RPS19-depleted cell lines with CoRPS19 LVs.

Two clinically applicable LVs carrying a codon-optimized version of the *RPS19* gene (*CoRPS19*) were generated. In the first one, *PGK.CoRPS19 LV*, the therapeutic gene was driven by the phosphoglycerate kinase (PGK) promoter. This promoter was preferentially selected since it could have regulatory advantages, due to its safety in several preclinical (28) and clinical studies, in particular in FA and pyruvate kinase deficiency trials. In the second, *EF1*α*.CoRPS19 LV*, the transgene was driven by the elongation factor alpha short [*EF1*α*(s)*] promoter (Supplemental Figure 2A). *EF1*α*.CoRPS19 LV* was constructed to compare with PGK, as an LV with this promoter had been previously tested in *RPS19* models (21, 23). The efficacy of these vectors was first tested in an *RPS19*-deficient DBA cell model generated by transducing K562 cells with 2 different interference LVs expressing an anti-*RPS19* shRNA sequence: *THM-sh LV* and *MISSION-sh LV*. The level of interference mediated by the former vector reduced *RPS19* mRNA levels to 30%–60% of the control level (left panel, Supplemental Figure 2B), mimicking the loss of function of *RPS19-*haploinsufficient cells. On the other hand, almost no *RPS19* expression was observed in *MISSION-sh* *LV*–transduced cells (left panel, Supplemental Figure 2C). This model mimicked mutations leading to a severe decrease in RPS19 expression (29). This resulted in a proliferative disadvantage compared with immortalized nontransduced K562 cells. Therefore, to ensure the selection of cells with a high percentage of transduction and high interference of endogenous *RPS19* expression, we sorted cells using the high-intensity Turbo EGFP marker, always ensuring we worked with cells with 95% fluorescence. Thereafter, transduction of either of these cell models with either of the therapeutic LVs promoted the expression of *CoRPS19* mRNA (right panels, Supplemental Figure 2, B and C), while neither therapeutic LV had any effect on the endogenous *RPS19* expression (left panels, Supplemental Figure 2, B and C).

**Figure 2:**
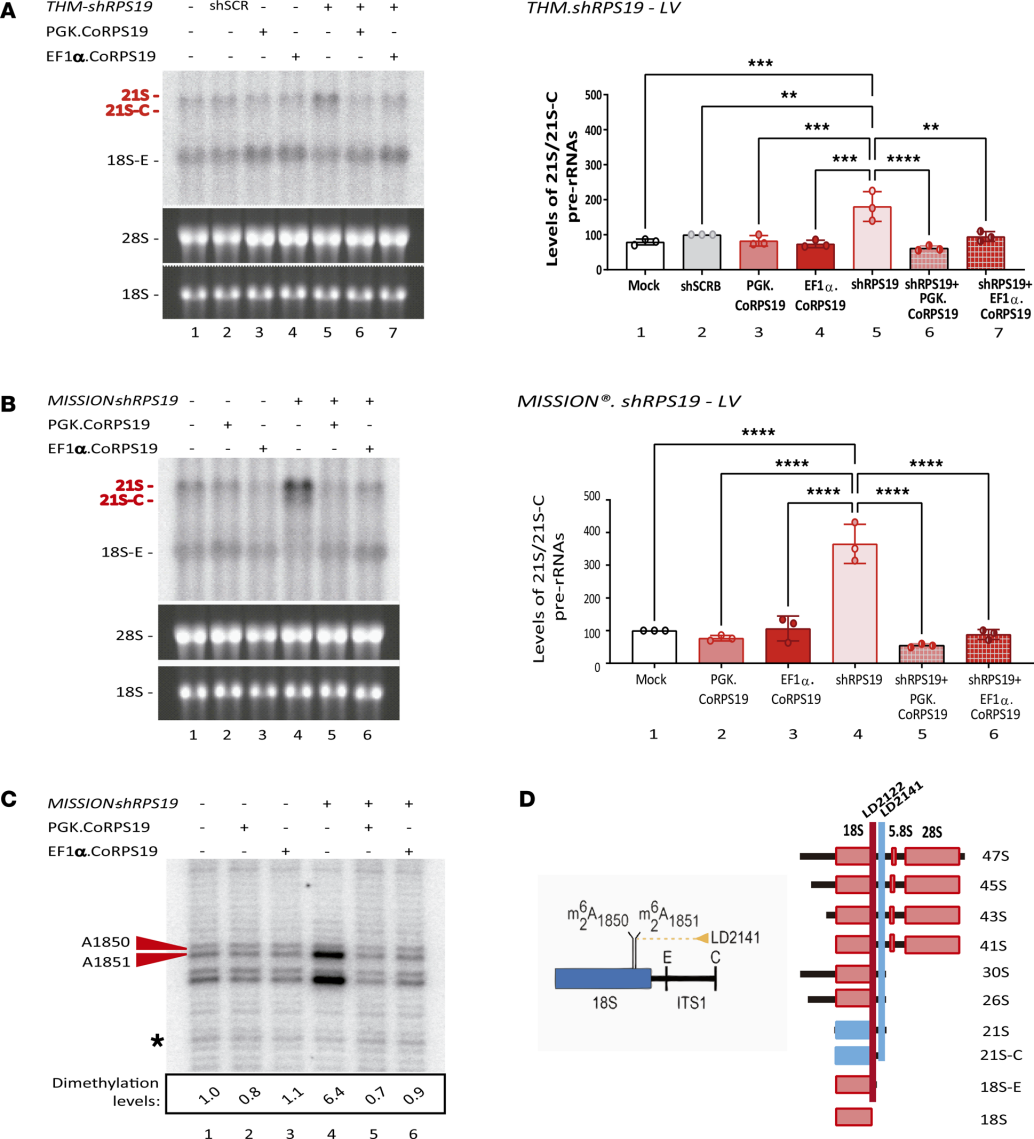
Phenotypic correction of DBA cell models using *PGK.CoRPS19 LV* and *EF1α.CoRPS19 LV* therapeutic LVs. (**A**) Northern blot analysis of precursor rRNA levels. Left, upper part: pre-rRNA precursors from K562 cells transduced with the interference vector *THM-shRPS19 LV*, with or without a therapeutic vector (*PGK.CoRPS19* or *EF1α.CoRPS19 LV*s), probed with a radioactively labeled oligonucleotide (LD2122). Left, lower part: mature 18S and 28S rRNAs on an ethidium bromide–stained gel. Right, phosphorimager quantification of the combined amounts of 21S and 21C pre-rRNAs, mean value and standard deviation of 3 experiments. The degree of significance was determined with the 1-way ANOVA test (*P* value; ** ≤ 0.01; *** ≤ 0.001; **** ≤ 0.0001). (**B**) Northern blot analysis of precursor rRNA levels. Left, total RNA extracted from K562 cells transduced with interference vector *MISSION-shRPS19 LV*, with or without the therapeutic *PGK.CoRPS19* or *EF1α.CoRPS19 LV*s. Right, phosphorimager quantification of the combined amounts of 21S and 21C pre-rRNAs, mean value and standard deviation of 3 experiments. The degree of significance was determined with the 1-way ANOVA test (*P* value; **** ≤ 0.0001). Both blots shown are representative of triplicate blots (Supplemental Figure 3). (**C**) Primer extension analysis of precursor rRNA dimethylation levels in K562 cells transduced with the interference vector *THM.shRPS19 LV*, with or without a therapeutic vector (*PGK.CoRPS19 LV* or *EF1*α*.CoRPS19 LV*). A radioactively labeled oligonucleotide, LD2122, specific to the internal transcribed spacer 1 (ITS1) sequence located 3′ to the dimethylation mark, was extended with reverse transcriptase (see **D**). The position of the dimethylation is indicated. Dimethylation levels were quantitated with a phosphorimager (signal normalized to the band denoted with a star) as described (31). (**D**) Schematics representing the oligonucleotides used in Northern blotting (LD2122) and primer extension (LD2141, see **C**) and the major pre-rRNA precursors detected.

These DBA-like K562 cells were then used to assess the efficacy of *PGK.CoRPS19* and *EF1*α*.CoRPS19 LV*s to correct the pre-rRNA processing inhibition. When these cells were not transduced with the therapeutic LVs, the ribosome biogenesis was compromised as a result of *RPS19* depletion, implying that the pre-rRNA processing was inhibited, leading to the accumulation of the 21S/21S-C precursors and to a reduced generation of 18S-E pre-rRNA, the direct precursor of 18S rRNA (Figure 2A, lane 5, and Figure 2B, lane 4). These effects of RPS19 depletion on the pre-rRNA processing are consistent with previous studies (30). As happened with the assessment of *RPS19* mRNA depletion by quantitative PCR (qPCR), the inhibition of the pre-rRNA processing was more pronounced with the *MISSION-shRPS19 LV* than with *THM-shRPS19 LV*.

When RPS19-interfered cells were then transduced with either therapeutic LV, the pre-rRNA processing was fully restored. This was deduced from the normalized levels of 21S/21S-C and 18S-E observed in these corrected cells (Figure 2A, lanes 6–7, and Figure 2B, lanes 5–6), suggesting that both therapeutic LVs are functional. As previously described (31), *RPS19* depletion also led to accumulation of the 41S pre-rRNA (see Figure 2D), which was also suppressed by expression of either *CoRPS19* *LV* (see Supplemental Figure 3).

At this stage, we sought to verify this outcome using a clinically applicable technique, suitable for small sample sizes, unlike Northern blotting, which necessitates 3–5 μg. We turned to an rRNA modification installed on 21S/21S-C and detectable with a sensitive primer extension assay. During ribosomal subunit biogenesis, the precursor rRNAs are extensively modified at specific positions by dedicated enzymes. This includes N^6,6^ dimethylation of 2 adjacent adenosines at the 3′ end of the 18S rRNA, performed in the nucleus by DIMT1L on the 21S/21S-C precursors (31). The 
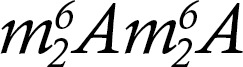
 modification can be detected by primer extension, and it interferes with reverse transcriptase progression, causing the enzyme to drop off its template so that the cDNA synthesized ends at the modified position. Quantifying this cDNA thus offers a proxy for precise evaluation of steady-state levels of 21S/21S-C by Northern blotting. A major advantage of the primer extension assay is that it is far more sensitive and requires as little as 500 ng input RNA.

In practice, we extended a primer, LD2141, specific to a sequence in the ITS1 located downstream of the dimethylation mark in the 21S/21S-C pre-rRNAs (Figure 2D). As expected, a low signal for dimethylation was detected in control cells (Figure 2C, lanes 1–3), corresponding to low levels of 21S/21S-C (Figure 2B, lanes 1–3; the same samples were analyzed in Figure 2, B and C). As predicted, this signal was strongly increased in cells depleted of *RPS19* (Figure 2C, lane 4), which is in line with the elevated levels of 21S/21S-C seen by Northern blotting (Figure 2B, lane 4). Cells expressing an anti-*RPS19* shRNA and transduced with either of the 2 therapeutic LVs displayed methylation levels similar to those of untreated cells (Figure 2C, compare lanes 5–6 with 1–3). This verified that both therapeutic LVs restore the processing function of RPS19. Additionally, we show a method that facilitates the analysis of 18S rRNA dimethylation for the diagnosis of *RPS19* deficiency–related processing defects, such as those typically observed in DBA, particularly when only limited amounts of RNA are available.

### Phenotypic correction of HSPCs from patients with RPS19 DBA with CoRPS19-bearing LVs.

**Figure 3:**
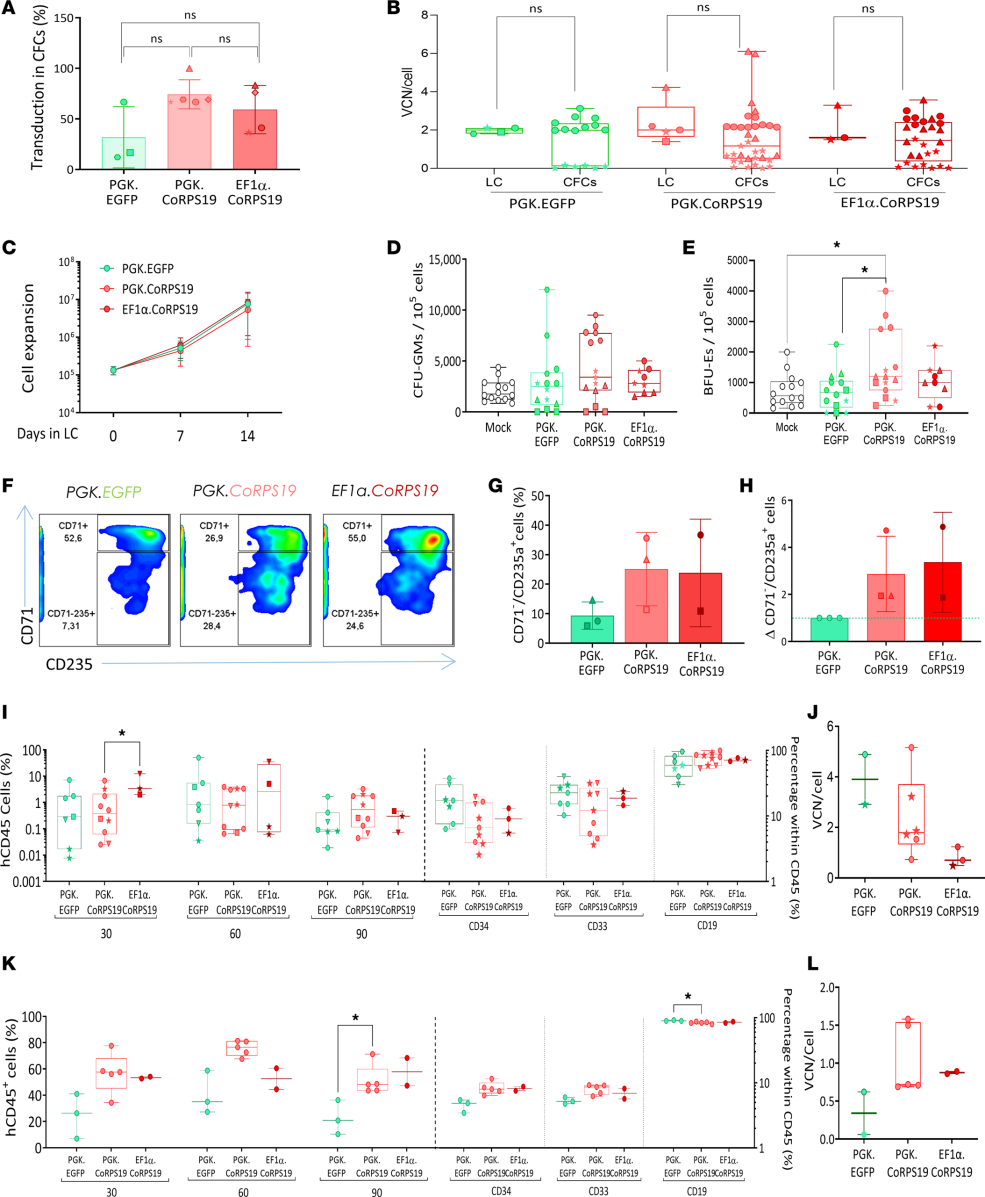
Phenotypic hematopoietic correction mediated by transduction of BM CD34^+^ cells from DBA patients with *PGK.CoRPS19 LV* or *EF1α(s).CoRPS19 LV*. (**A**) Transduction efficiency of DBA patient hematopoietic progenitors: *PGK.EGFP LV* (*n* = 4), *PGK.CoRPS19 LV* (*n* = 6), or *EF1*α*.CoRPS19 LV* (*n* = 4). The data are presented as means with standard deviation. CFCs, colony-forming cells. (**B**) Vector copy number (VCN) in cells maintained in liquid culture (LC) and in CFCs at 14 days of culture. *PGK.EGFP LV* (*n* = 4), *PGK.CoRPS19 LV* (*n* = 5), and *EF1α.CoRPS19 LV* (*n* = 3). (**C**) Number of HSPCs after 0, 7, and 14 days of LC expansion specific for HSCs. (**D** and **E**) Number of CFU-GM and BFU-E colonies per 10 × 10^–5^ mononuclear cells seeded. *n* = 3 *PGK.EGFP LV*, *n* = 5 *PGK.CoRPS19* *LV*, and *n* = 5 *EF1*α*.CoRPS19* *LV*. (**F**) Flow cytometry strategy used to analyze erythroid progenitors, (**G**) percentage of CD71^–^/CD235a^+^, and (**H**) increment of CD71^–^/CD235a^+^ mature erythroid progenitors, obtained in BM CD34^+^ DBA cells from 3 patients posttransduction at day 14 of specific erythroid expansion culture. The graph shows the mean and standard deviation obtained in 3 patients. (**I**) Left: percentage of hCD45^+^ engrafted cells at 30, 60, and 90 days posttransplantation with 10 × 10^–5^ BM CD34^+^ transduced cells from DBA patients. Right: myeloid cells (CD33^+^), lymphoid cells (CD19^+^), and HSCs (CD34^+^) of DBA patient cells transplanted posttransduction into NSG mice. Each symbol represents 1 specific patient. (**J**) VCN at day 90 posttransplantation. The graph shows the mean and standard error of the mean. (**K**) Left: Percentage of hCD45^+^ cells measured 30, 60, and 90 days (indicated below the graph) after transplantation of 8 × 10^–5^ BM CD34^+^ cells from patient DBA-37. Right: Myeloid cells (CD33^+^), lymphoid cells (CD19^+^), and HSCs (CD34^+^) of DBA patient cells transduced and transplanted into NSG mice. (**L**) VCN, at day 90 posttransplantation, in hCD45^+^ cells (from patient DBA-37). Multiple-comparison Kruskal-Wallis test (*P* value; * ≤ 0.017).

To investigate the efficacy of the therapeutic LVs to correct the phenotype of HSCs from *RPS19-*mutated DBA, BM CD34^+^ cells from these patients were transduced with either therapeutic vector or with a control LV (*EGFP LV*) (Table 1). As shown in Figure 3A and Table 2, both therapeutic LVs transduced at a similar efficacy the CFCs of patients with DBA. Consistently, the VCN per cell was similar in the different groups of transduced CD34^+^ cells, regardless that analyses were performed on cells from liquid cultures or from individual colonies (Figure 3B). CD34^+^ cells transduced with either LV showed comparable growth kinetics in liquid cultures (Figure 3C), indicating that the therapeutic LVs neither conferred obvious proliferative advantage nor exerted significant toxicity as compared to the control LV. Similarly, clonogenic assays showed that DBA patient CD34^+^ cells transduced with either therapeutic LV or with the control LV generated similar numbers of CFU-GMs (Figure 3D). In contrast with this observation, BFU-E numbers were significantly increased in cells that had been transduced with the *PGK.CoRPS19 LV* (Figure 3E).

Since a hallmark of patients with RPS19-mutated DBA is their ineffective erythropoiesis, CD34^+^ cells from 3 patients were transduced with either LV and then allowed to differentiate toward the erythroid lineage in liquid culture. As shown in Figure 3, F–H, although the increase was not significant, the transduction of these cells with the therapeutic LVs increased the percentage of mature CD71^–^ and CD235a^+^ erythroid cells 2.8- and 3.4-fold, respectively, as compared with the control samples (same CD34^+^ cells transduced with *EGFP LV*).

Next, we studied the in vivo effects associated with the transduction of *RPS19*-mutated DBA patient CD34^+^ cells with the therapeutic LVs; 100,000 CD34^+^ cells transduced with either LV were transplanted into immunodeficient NSG mice. Analysis of percentage of human CD45^+^ cells in the BM of recipient mice showed similar levels of human hematopoietic repopulation in all 3 groups of transplanted mice, apart from a significant increase in the *EF1*α*.CoRPS19* group observed on day 30 posttransplantation (Figure 3I). No differences among the 3 groups of transplanted mice were observed in the proportions of HSPCs (CD34^+^), myeloid cells (CD33^+^), or B lymphoid cells (CD19^+^) (Figure 3I). Analysis of VCN in recipient BM samples showed mean values of 3.90 ± 0.99, 2.37 ± 0.65, and 0.81 ± 0.22 copies per human genome in the *PGK.EGFP*, *PGK.CoRPS19*, and *EF1*α*.CoRPS19* groups, respectively (Figure 3J). An additional experiment was conducted in which it was possible to infuse 800,000 transduced *RPS19*-mutated DBA patient CD34^+^ cells per mouse. In comparison with the *PGK.EGFP* control group, a higher percentage of human CD45^+^ cells was observed in the *PGK.CoRPS19* group at 90 days posttransplantation, again with similar contributions to the different hematopoietic lineages (Figure 3K). The presence of the therapeutic vector in a high proportion of engrafted human cells (1.04 ± 0.2 and 0.88 ± 0.02 proviral copies/human genome in recipients of *PGK.CoRPS19 LV* and *EF1*α*.CoRPS19 LV*, respectively) verified the long-term repopulation capacity of transduced DBA HSPCs in vivo (Figure 3L).

**Figure 4:**
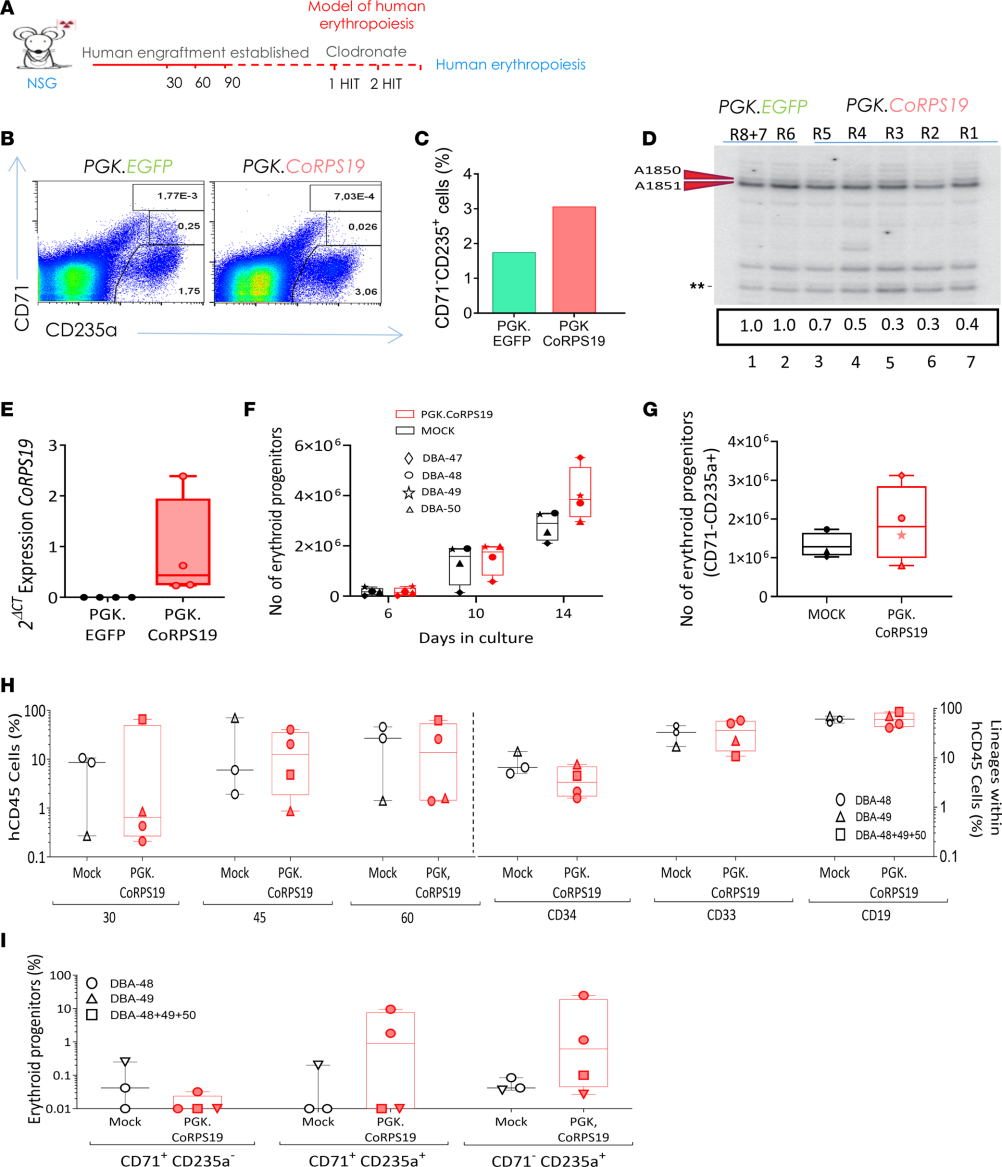
Improved erythroid differentiation mediated by transduction of BM CD34^+^ cells from DBA patients with *PGK.CoRPS19 LV* or *EF1*α*(s).CoRPS19 LV*. (**A**) Protocol used to obtain a model of in vivo erythropoiesis in immunodeficient NSG mice. (**B**) Flow cytometry strategy used. (**C**) Bar plot showing the percentage of CD71^–^/CD235a^+^ cells resulting from BM hCD45^+^ DBA cells transduced with *PGK.CoRPS19* *LV*. (**D**) Primer extension analysis of precursor rRNA dimethylation levels. The experiment was performed on human CD45^+^ hematopoietic cells purified at 90 days posttransplantation from NSG mice that had been transplanted with DBA patient CD34^+^ cells previously transduced with the therapeutic vector *PGK.CoRPS19 LV*. Arrows indicate the position of the dimethylation. Dimethylation levels were quantitated (signal normalized to the band denoted with a double star). (**E**) Expression of CoRPS19 in RPS19-deficient CD34^+^ cells corrected with *PGK.CoRPS19* *LV*, after 14 days of expansion in erythroid differentiation medium. The graph shows the mean and the standard deviation (*n* = 4). (**F**) Erythroid progenitor expansion in erythroid differentiation medium after transduction of thawed CD34^+^ cells from 4 DBA patients with *PGK.CoRPS19 LV*, as compared with the corresponding nontransduced RPS19-deficient cells (MOCK). (**G**) Total yield of CD71^–^/CD235a^+^ mature erythroid progenitors after transfection of thawed CD34^+^ cells with *PGK.CoRPS19* *LV*, as compared with nontransduced RPS19-deficient cells (mock). The graph shows the mean and the standard deviation. (**H**) Left: Engraftment level (percentage of hCD45^+^ cells) measured 30, 45, and 60 days after transplantation of 3 × 10^–5^ BM CD34^+^ cells from 4 DBA patients (DBA-47, DBA-48, DBA-49, DBA-50) in NBSGW mouse strain. Right: multilineage potential of DBA patient cells transduced with the therapeutic vector *PGK.CoRPS19 LV* or untransduced: myeloid cells (CD33^+^), lymphoid cells (CD19^+^), and HSCs (CD34^+^). Each symbol represents 1 specific patient. (**I**) Engrafted erythroid cell populations (expressed as percentages) observed at 45 days posttransplantation in *NBSGW* recipients.

To analyze the effects of GT on human erythroid differentiation in vivo, 1 mouse from the control group and 1 from the therapy group were treated with clodronate liposomes to deplete the splenic and BM macrophages and thus enhance human erythropoiesis (Figure 4, A and B). In this case we could observe that transduction with *PGK.CoRPS19 LV* increased the percentage of CD235a^+^CD71^–^ cells from 1.75 to 3.06, thus revealing a marked improvement in the in vivo erythroid differentiation of cells from a patient with DBA (Figure 4C).

Next, since ribosome biogenesis is deficient in RPS19-mutated cells, we investigated whether *PGK.RPS19 LV* could correct this defect in vivo. These analyses were focused on the *PGK.CoRPS19 LV* because of the more consistent results observed with this vector and of the fact that PGK-based LVs have been already used in several preclinical and clinical GT trials, including FA, without any safety concern (32–36). We thus transplanted DBA patient CD34^+^ cells that had been transduced with either the *PGK.EGFP LV* (control group) or the *PGK.RPS19 LV*. Given the limited number of human hematopoietic CD45^+^ cells purified from the BM of recipient mice at 90 days posttransplantation, we used the primer extension assay described in Figure 2C to estimate levels of 21S/21S-C pre-rRNA. Compared with samples collected from the control group (Figure 4D, lanes 1 and 2), a reduced dimethylation status was observed in each of the 5 samples obtained from mice engrafted with cells transduced with the therapeutic vector. This indicates functional complementation of the *RPS19* deficiency by *PGK.CoRPS19 LV* (Figure 4D, lanes 3–7).

Finally, since the NSG model is not ideal for evaluating human erythropoiesis, another set of experiments was performed in *NOD.Cg-KitW-41J Tyr 1 Prkdc scid Il2rgtm1Wjl* (NBSGW) mice, in order to evaluate the effect of GT in recipients with higher levels of human erythroid cells. In these experiments, CD34^+^ cells purified from frozen MNCs from 4 different *RPS19*-deficient patients were transduced with the *PGK.CoRPS19 LV*. Thereafter, an aliquot of these cells was maintained in liquid cultures and another one transplanted into *NBSGW* mice. As shown in Supplemental Figure 4, A and B, a high proportion of cells was transduced with the *PGK.CoRPS19 LV* (transduction efficacy: 71.4% ± 13.8% of CFCs; 2.2 ± 0.2 vector copies/cell). Compared with mock cells, cells transduced with the therapeutic LV expressed substantial levels of *CoRPS19* (Figure 4E) and showed a slight, nonsignificant increase over the control group either in HSC liquid cultures (Supplemental Figure 4C) or in cultures promoting specific growth of erythroid cells (Figure 4, F and G). In these cultures, a higher output of mature erythropoietic progenitors (CD71^–^CD2352a^+^) was observed, which, however, did not reach statistical significance (Figure 4, F and G). Subsequently, in analyses in NBSGW mice, we observed similar levels of engraftment in DBA CD34^+^ cells regardless of whether these cells were gene-corrected (Figure 4H). Nevertheless, generalized higher levels of erythroid progenitors were observed in recipients who had been transplanted with *PGK.CoRPS19* *LV*s (Figure 4I).

These studies thus indicated that transduction of *RPS19* patient CD34^+^ cells with therapeutic LVs preserves the hematopoietic repopulation of their HSCs and improves the erythroid differentiation of these cells in vitro and in vivo.

### Safety aspects associated with the therapeutic vector PGK.CoRPS19 LV.

**Figure 5:**
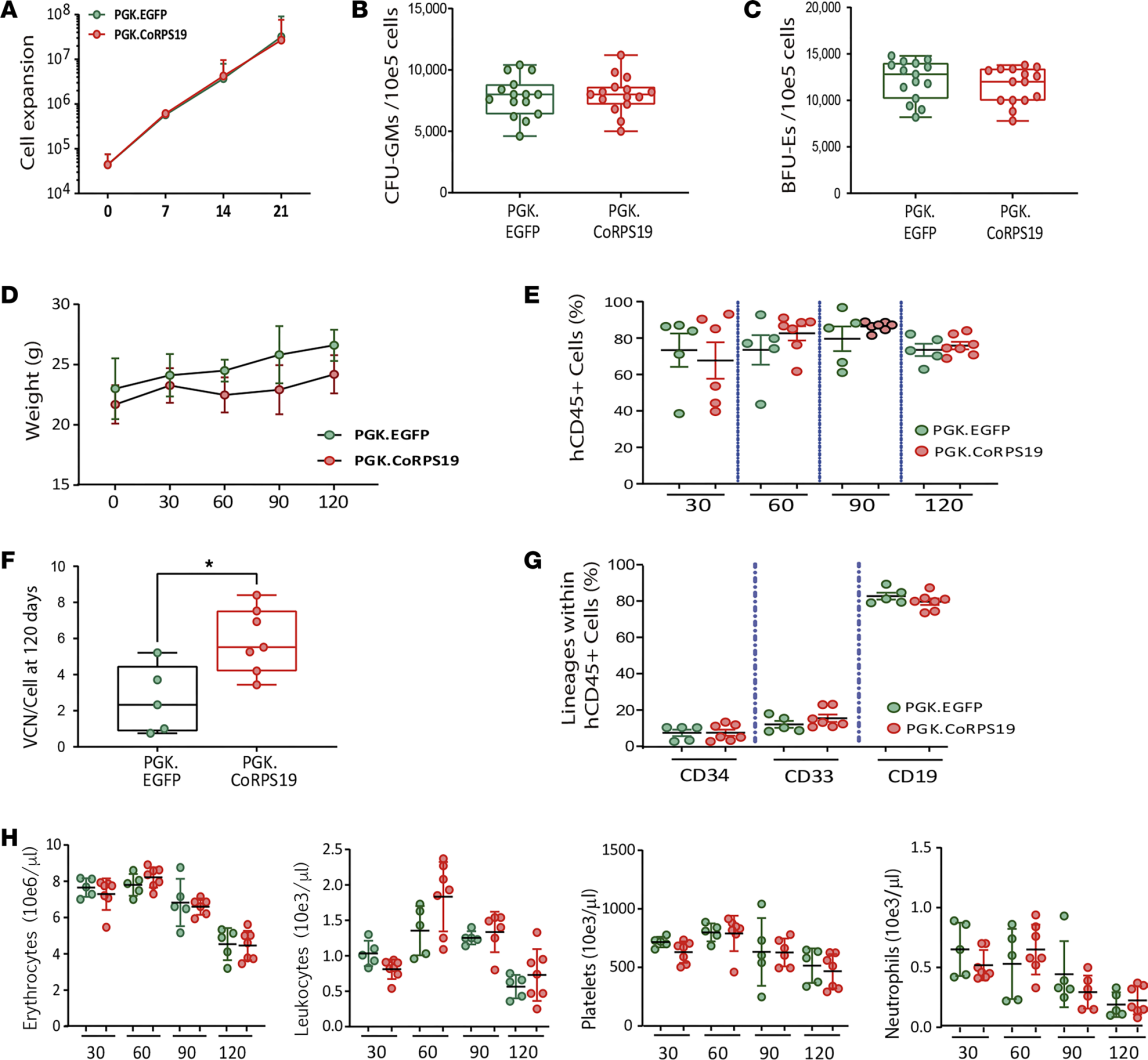
Safety studies mediated by the overexpression of RPS19 in cord blood CD34^+^ cells from HDs transduced with the therapeutic *PGK.CoRPS19* LV. Graphs refer to HD cord blood (CB) CD34^+^ cells transduced with therapeutic vector *PGK.CoRPS19 LV* or the control vector *PGK.EGFP LV*. (**A**) Growth curves of HD HSPC progenitors maintained in HSC expansion medium, after transduction. The graph shows the mean and standard deviation of 5 experiments. Mann-Whitney test. *x* axis, days. (**B** and **C**) Healthy donor umbilical cord CD34^+^ cells transduced with *PGK.CoRPS19 LV* or *PGK.EGFP LV* (control). (**B**) Number of granulocyte-macrophage progenitor colony-forming units (CFU-GMs) per 10^5^ mononuclear cells seeded. (**C**) The number of BFU-Es per 10^5^ mononuclear cells seeded. The degree of significance was determined with the Mann-Whitney test. (**D**) Body weight of NSG mice transplanted with HD CD34^+^ cells transduced with the therapeutic vector *PGK.CoRPS19 LV* or the control vector *PGK.EGFP LV*. (**E**) The repopulation potential was determined by quantifying the percentage of hCD45^+^ cells in NSG recipient mice up to day 120. (**F**) VCN in human HSPC engrafted into NSG recipient mice. The graph shows the mean and standard error of the mean of 2 different experiments after transducing healthy donor CD34^+^ cells from CB and transplanting them into NSG mice. Statistical significance was determined with the Mann-Whitney test (*P* value; * ≤ 0.05). (**G**) Distribution of myeloid cells (CD33^+^), lymphoid cells (CD19^+^), and HSCs (CD34^+^) in NSG recipients. (**H**) Determination of hematological parameters in NSG mice transplanted: erythrocytes, leukocytes, platelets, and neutrophils. The graph shows the mean and standard error of the mean of 2 different experiments conducted to analyze safety and in vivo toxicity effects of the therapeutic vectors after transfecting HD CD34^+^ cells from CB and transplanting them into NSG mice. Statistical significance was determined with the 2-tailed Mann-Whitney test. *x* axis, days.

Next, we investigated the possibility that the induction of supraphysiological levels of *RPS19* might lead to toxic effects in human HSPCs. To investigate this, we focused on the *PGK.CoRPS19 LV* because of previous results and our preference from a regulatory perspective. Cord blood CD34^+^ cells from HDs were transduced with the *PGK.CoRPS19 LV* and the *PGK.EGFP* *LV* and then tested in vitro and in vivo (Supplemental Figure 5). When samples were assessed in liquid and methylcellulose cultures, both the cell growth kinetics (Figure 5A) and the CFU-GM and BFU-E counts (Figure 5, B and C) were similar in samples that had been transduced with the EGFP and the RPS19 LVs. With respect to the in vivo studies, when CD34^+^ cells transduced with either of these LVs were infused into immunodeficient NSG mice, the 2 groups of mice showed similar body weight evolution posttransplantation (Figure 5D). Additionally, and consistently with the in vitro data, no significant differences were observed in the repopulating potential of cells transduced with the control or the therapeutic LV (Figure 5E), even though the VCN/cell in this experiment was particularly high in recipients infused with the therapeutic LV (Figure 5F). Once again, no differences in the differentiation lineages of the engrafted cells were noted between the 2 groups (Figure 5, G and H).

**Figure 6:**
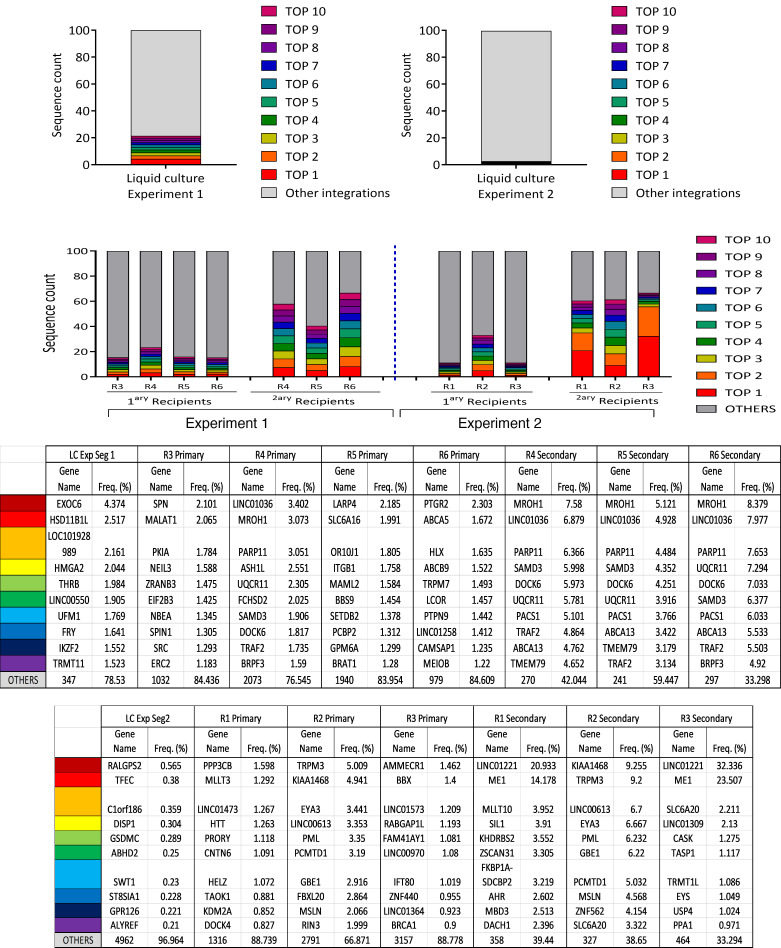
Analysis of the ISs of *PGK.CoRPS19 LV* in CD34^+^ cells from HDs. The data are presented as cumulative retrieval frequencies of the 10 most prominent cell ISs detected in liquid cultures and all mouse recipient samples (referred to as R1 to R6). For individual samples, sequence data from all shearing extension first tag selection ligation-mediated PCR replicates were combined. Sequence counts for the 10 most prominent ISs, of all remaining ISs as well as total sequence count from all replicates are shown at the bottom for each sample. RefSeq names of genes located closest to ISs are in the table. Relative sequence count contributions of the 10 most prominent ISs and of all remaining mappable ISs are shown (frequency).

Last, we analyzed common integration sites (CISs) in recipients transplanted with samples transduced with therapeutic *PGK.CoRPS19* *LV*. When transduced samples were analyzed in liquid cultures prior to transplantation, the contribution of the clone with the highest representation in the cell culture was as low as 4.37% (Figure 6). Similarly, the clone with the highest contribution in transplanted NSG recipients did not exceed 5.0% of the total clonal repertoire, revealing the diversity of the clonal repertoire of engrafted hematopoiesis. In secondary recipients, the strongest contribution of an individual IS was observed in a sample of a secondary recipient with a relative contribution of 32.336%. CIS analysis revealed a diverse composition of CISs, and none of the top 10 CIS regions has been associated to adverse events like clonal outgrowth or malignant transformation in clinical GT trials reported to date (Figure 6). Consistent with these observations, no expansion of clones with specific integrations into or near genes implicated in genotoxic events induced by gammaretroviral vectors (*CCND2*, *LMO2*, *MDS1/EVI1* [*MECOM*], *MN1*) was observed (Supplemental Figure 6). These experiments demonstrate that transplantation of CD34^+^ cells transduced with the therapeutic *PGK.CoRPS19 LV* was associated with a safe polyclonal repertoire, with no evidence of genotoxic insertions.

## Discussion

While HSCT remains the only curative option for patients with DBA, the risks of transplant-related mortality and of graft-versus-host disease of these patients increase with the age, limiting the applicability of this therapeutic intervention. Additionally, patients with *RPS19*-mutated DBA are characterized by a poor response to steroids, the standard treatment for DBA (1, 37, 38), and have a poorer long-term prognosis as compared with other DBA patients (39), indicating the necessity of developing safe and efficient therapies for these patients.

As in other monogenic diseases, GT may circumvent several HSCT-related complications, providing a good alternative for patients who are not eligible for HSCT. Although previous elegant experimental studies showed the efficacy of GT to correct some phenotypic signs of *RPS19* deficiency, there is a lack of preclinical evidence demonstrating the safety and efficacy of clinically applicable vectors in HSCs from patients with DBA. Previous studies with clinically applicable vectors have been limited to the use of DBA mouse models (21–23) or to the evaluation of the response of RPS19-interfered HSCs from HDs (23, 24), which do not accurately replicate the HSC defects characteristic of patients with DBA.

Data presented in this study show the safety and efficacy of a GT approach using primary DBA patient HSCs that had been transduced with clinically applicable LVs, bringing GT for this disease one step closer to clinical use. Although the overall clinical manifestations of patients with DBA are mainly manifested in the erythroid lineage, it has been proposed that defects in rRNA processing, and probably also the chronic anemia characteristic of the disease, might trigger HSC exhaustion, potentially leading to proliferative HSC defects in these patients (40).

In the particular case of FA, previous studies have revealed that one of the limiting steps in the development of FA GT is the low number of HSCs than can be collected from patients at a relatively advanced stage of the disease. For this reason, collection of clinically relevant numbers of CD34^+^ cells is currently restricted to patients younger than the age of 10 and with a threshold of number of 30 CD34^+^ cells/μL in BM (16). Despite this limitation, the strong proliferative advantage of the FA-corrected HSCs initially observed in preclinical studies (14) was thereafter reproduced in patients with FA infused with corrected autologous HSCs, even in the absence of any conditioning regimen (13).

In the current study, we reveal that in contrast with FA, DBA patients have a markedly higher CD34^+^ cell content. Moreover, as deduced from our flow cytometry studies, numbers of HSPCs determined in patients with DBA are similar to those observed in HDs, though with some myeloid skewing. Additionally, clonogenic studies show that compared with HD BM samples, DBA patient BM samples contained a modest reduction in CFU-GM numbers and a significant reduction in BFU-Es, in good consistency with previous studies (41–44). Once again, these defects were markedly more severe in BM samples from FA. Additionally, our transplantation experiments in NSG mice show that uncorrected DBA CD34^+^ cells maintained the in vivo repopulating ability, even though analyses conducted at 90 days postinfusion showed a marked reduction compared with data obtained from HDs. Although these results are consistent with previous data suggesting that HSCs from DBA do not behave like healthy HSCs (45, 46), DBA HSCs clearly differ from FA HSCs, since these precursors only showed significant repopulation ability after gene complementation (13, 14).

Our analyses of DBA HSPCs are consistent with previous studies showing that most clinical signs of DBA are mainly evident in committed progenitors and mature cells of the erythroid (43, 47, 48) and megakaryocytic (27) lineages, though certain defects, characteristic of inflammatory hematopoiesis and of hematopoiesis in patients who are elderly (16), may arise even at the HSPC level (45, 46).

In relation to GT, it is remarkable that the baseline defects observed in HSCs from DBA are much less pronounced than those observed in their FA counterparts, suggesting that collection of HSCs from DBA should not constitute a significant restriction, at least in the age range of the patients analyzed here, all of whom were below 50 years of age.

Using 2 DBA cell models (K562 cells with an interfered expression of RPS19 by different shRNAs), we demonstrated that the therapeutic vectors generated in our study can effectively complement the ribosomal biogenesis defects caused by the reduced expression of RPS19. We have also shown in CD34^+^ cells from patients with *RPS19*-deficient DBA and treated or not with the *PGK.CoRPS19* *LV* that transduction with *PGK.CoRPS19* *LV* significantly increases the clonogenic potential of the erythroid progenitor cells (BFU-Es) (*P* ≤ 0.017) and improves the characteristic ineffective erythropoiesis from DBA.

Our transplantation experiments showed that corrected CD34^+^ cells from patients with DBA sustained their repopulation potential and that engraftment of *PGK.CoRPS19*-corrected HSCs should bypass the erythroid deficiency and abortive ribosome biogenesis characteristic of DBA cells. However, our findings suggest that in marked contrast with FA, or even with data previously obtained with the *Rps19* model (22) or after transduction with onco-retroviral vectors (26), there is not a marked proliferative advantage in corrected HSCs from DBA with respect to uncorrected HSCs. Although in some specific patients or cases where a high number of cells could be infused, there might be an increased repopulation capacity of *RPS19* HSPCs after genetic correction, a marked repopulating potential is not expected when these cells are infused back into the patient.

Likewise, the cases of spontaneous hematologic improvement due to somatic mosaicism are rarely described in DBA (26, 47) compared with FA, and it remains controversial whether the reversion occurred in primitive HSCs or only in the compromised erythroid progenitor cells.

Consequently, these data are indicative of the desirability of treating patients with DBA on GT with at least moderate or submyeloablative conditioning regimens prior to infusion of transduced HSCs.

While Northern blotting constitutes the classic, gold standard method for assessing pre-rRNA processing, used extensively in the context of DBA research, it is somewhat limited by the large amount of material (total RNA in the microgram range) required to conduct these studies. Such amounts can hardly be obtained from purified hematopoietic cells, hence the need to develop more sensitive assays. To start addressing this, we endeavored to quantitate the 18S rRNA dimethylation installed during ribosomal subunit biogenesis at the level of 21S/21S-C pre-rRNA as a proxy for measuring 21S/21S-C levels directly. We validated this idea by efficiently detecting this dimethylation in a sensitive primer extension assay and by showing that the dimethylation signal correlates very well with 21S/21S-C pre-rRNA abundance. We further used this assay successfully on low-input RNA extracted from purified hematopoietic human cells to demonstrate that LVs expressing the therapeutic gene from the PGK promoter functionally complements defects caused by the RPS19 loss.

Last, the safety studies conducted in this comprehensive work include a systematic analysis of genomic ISs, which provided no evidence of toxicity. These results are consistent with those obtained in previous studies in which self-inactivating vectors containing eukaryotic promoters were used (12) and also with studies showing that the potential overexpression of RPs is downregulated through proteasomal degradation (22).

GT is a rapidly evolving field, and gene editing (GE) is already being investigated, including in FA (49). However, a single approach does not cover the full spectrum of mutations, and the delivery methods for HSCs remain challenging. Additionally, the safety aspects of GE have not been fully investigated. It may not be possible to correct the wide range of genetic defects, such as substitutions, extensive genomic deletions, and frameshifts, identified in RPS19 or other ribosomal protein genes or assembly factor genes causing DBA using DSB-free GE techniques. On the other hand, if a transduced RPS19 gene is expressed from a PGK promoter, it may allow many RPS19-deficient patients who are currently ineligible for HSCT to receive treatment. This treatment would restore sufficient RPS19 function in all HSPCs where decreased expression has a significant effect (24, 50, 51).

In conclusion, the preclinical studies conducted in this work strongly suggest that LV-mediated GT, specifically with *PGK.CoRPS19 LV* (for which the orphan drug designation “EMA/OD/0000060656” has been obtained), should provide an efficient and safe approach for correcting the hematological defects characteristic of *RPS19*-deficient DBA.

## Methods

### Sex as a biological variable

Sex was not considered a biological variable in samples from HDs or patients with DBA since no differences have been described in the frequency of the disease. Only female mouse recipients were used for transplant because of improved human engraftment compared with males in immunodeficient models.

### LV production

Therapeutic vectors were generated from the previous LV pCCL.sin.ppt.hPGK.EGFP.Wpre* vector provided by Luigi Naldini (San Raffaele Telethon Institute for Gene Therapy and Vita Salute, San Raffaele University Medical School, Milan, Italy). The first of the interference LVs was generated cloning the sequence 5′GATCCCCGTCCGGGAAGCTGAAAGTCTTCAAGAGAGACTTTCAGCTTCCCGGACTTTTTGGAA3′ previously described (52) in the lentiviral skeleton *LV-THM.sh7FANCA* provided in-house**.**

The second LV, LV-MISSION pLKO.1-pure TurboGFP shRPS19, was a commercial product (TRCN0000074915, NM_001022.3-403s1c1, Merck) described before (53). Lentiviruses were produced as previously described (54). For LVs’ titration, 5 × 10^4^ HEK293T cells were transduced; 14 days after transduction, the number of LV genomes (VCN) integrated into transduced cells was determined by qPCR according to Charrier et al. (55). All reactions were carried out in duplicate in a 7500 Fast Real-Time PCR System (Applied Biosystems, Thermo Fisher Scientific).

### Cell line cultures

The K562 cell line (chronic myelogenous leukemia; ATCC: CCL-243) was grown in Iscove’s modified Dulbecco’s medium (IMDM; Gibco, Thermo Fisher Scientific), HyClone (10%; GE Healthcare, now Cytiva), and penicillin/streptomycin (1%; Gibco, Thermo Fisher Scientific) at a concentration of 1 × 10^5^ to 1 × 10^6^ cells/mL. The HEK293T cell line (human embryonic kidney cell line competent to replicate vectors bearing the SV-40 T antigen; ATCC: CRL-3219) was grown in IMDM, HyClone, and penicillin/streptomycin at a concentration of 5 × 10^5^ cells/mL. The incubation conditions were 37°C, 5% CO_2_, and 95% relative humidity. For transduction 2 × 10^5^ K562 cells were seeded and transduced at MOI 25, 2 times on consecutive days. Transduction efficiency was checked by flow cytometry.

### Hematology analyses and transduction of human hematopoietic progenitors

Hematology analyses from samples from patients with DBA and HDs were performed on the Sysmex XN-1000 hematology analyzer from 120 μL of peripheral blood or BM in pre-dilution mode.

For the transduction, MNCs were obtained by Ficoll-Paque PLUS (GE Healthcare) density gradient isolation according to the manufacturer’s recommendations. Purified CD34^+^ cells were obtained by immunoselection using the CD34 Micro-Bead Kit (MACS; Miltenyi Biotec) according to the manufacturer’s recommendations. Fresh or thawed BM-CD34^+^ cells were prestimulated and transduced as previously described (56) in hypoxic conditions (5% O_2_) with the therapeutic PGK.CoRPS19.Wpre*, therapeutic *EF1*α*(s).CoRPS19.Wpre*, and control PGK.EGFP.Wpre* LVs for 16 ± 4 hours. After transduction, cells were washed and seeded for clonogenic assays, in vitro expansion for 14 days, or transplantation.

### Determination of VCN in hematopoietic progenitors obtained in liquid culture and clonogenic culture

VCNs were determined as previously described (14, 55) in cells expanded for 14 days in liquid culture, in clonogenic cultures maintained during 14 days, and in the human engrafted cells in mice at different time points.

### Determination of CoRPS19 expression

The expression of the CoRPS19 transgene integrated, as well as its nonoptimized physiological version RPS19, was carried out by qPCR using primers specific for h*GAPDH* Fw:GCTCTCTGCTCCTCCTGTTC, h*GAPDH* Rv:ACGACCAAATCCGTTGACTC, *RPS19* Fw:AGCCGAGGCTCCAAGAGT, *RPS19* Rv:CCTGAGGTGTCAGTTTGC, *CoRPS19* Fw:GTGAACCAGCAGGAGTTCGT, and *CoRPS19* Rv:CAGCTTGCCGCTCTTCT. RNA was extracted from cells using the RNeasy Tissue kit (QIAGEN GmBH), and cDNA was transcribed with Invitrogen SuperScript IV VILO Master Mix (Thermo Fisher Scientific). Expression analysis was in a 7500 real-time PCR system (Thermo Fisher Scientific). Expression of the endogenous *RPS19* gene, and CoRPS19, was calculated with a variation of the method of Livak (2ΔCt). To this end, the amplification efficiency of these genes and the h*GAPDH* was determined.

### Northern blotting and primer extension analysis of pre-rRNA processing

Total RNA was extracted using the TRI reagent (Invitrogen, Thermo Fisher Scientific), and 5 μg was either resolved on a denaturing agarose gel and processed for Northern blotting with probe LD2122 (GCCCTCCGGGCTCCGTTAATGATC), as described in Tafforeau et al. (57), or analyzed by primer extension to detect the dimethylations at the positions A1850 and A1851 of the 18S rRNA with oligo LD2141 (CGAGCGAGCGAACGAACGGGC), as described (58). The Northern blots and primer extension assays were exposed to Fuji imaging plates (Fujifilm), and quantification was performed on a phosphorimager (FLA-7000; Fujifilm) using the MultiGauge software (Fujifilm, v 3.1).

### Hematopoietic characterization of patients with DBA

#### Immunophenotype of patients with DBA and HDs.

The immunophenotype of patients with DBA and HDs was analyzed by flow cytometry using panels specified in Supplemental Table 1. Lysis with ammonium chloride buffer (0.155 M NH_4_Cl, 0.01 M KHCO_3_, 0.1 mM EDTA; 10-minute incubation at room temperature) was performed on panels 3, 4, 5, and 6. DAPI was added to a final concentration of 1 μg/mL to identify dead cells. The determination of the different hematopoietic progenitors in BM of patients with DBA and HDs was carried out using the antibodies reflected in Supplemental Table 2. The events were acquired and recorded with a Fortessa LSR (BD Biosciences) and analyzed by FlowJo software (BD Biosciences).

#### Hematopoietic progenitor and HSC functional assays.

CD34^+^ cells were purified from peripheral blood and BM samples from patients with DBA and HDs for characterization and lentiviral correction studies and from umbilical cord blood from HDs for safety studies.

CD34^+^ cells were cultured in StemSpam medium (StemCell Technologies) or X-VIVO 20 medium (Lonza) supplemented with 1% GlutaMAX (Gibco, Thermo Fisher Scientific), 1% penicillin/streptomycin, 100 ng/mL hSCF and hFlt3, and 20 ng/mL human thrombopoietin and hIL-3 (all EuroBioSciences GmbH) under hypoxic conditions (37°C, 5% O_2_, 5% CO_2_, and 95% relative humidity).

#### Erythroid differentiation protocol of human hematopoietic progenitors.

Human hematopoietic progenitors were differentiated toward the erythroid lineage using 3 different steps. The first medium (day 1–7) was based on StemSpan SFEM I (StemCell Technologies) supplemented with hSCF (50 ng/mL), hFlt3-ligand (16.7 ng/mL; EuroBioSciences), bone morphogenetic protein 4 (6.7 ng/mL; PeproTech), hIL-3 (6.7 ng/mL; EuroBioSciences), hIL-11 (6.7 ng/mL; EuroBioSciences), and human erythropoietin (hEPO; 1.3 U/mL; Amgen). On day 7, the cells were transferred to the second medium composed of IMDM supplemented with glutamine (supplemented with IMDM GlutaMAX; Gibco, Thermo Fisher Scientific) and enriched with BSA (1%; MilliporeSigma), insulin (0.01 mg/mL; MilliporeSigma), human transferrin (0.2 mg/mL; MilliporeSigma), β-mercaptoethanol (91 μM; Gibco, Thermo Fisher Scientific), penicillin/streptomycin (1%), lipid mix 1 (1×; MilliporeSigma), ethanolamine (0.004%, MilliporeSigma), hSCF (5 ng/mL; EuroBioSciences), hIL-3 (6.7 ng/mL; EuroBioSciences), hIL-11 (6.7 ng/mL; EuroBioSciences), hEPO (1.3 U/mL; Amgen), IGF-1 (20 ng/mL; PeproTech), and hydrocortisone (1 μM; MilliporeSigma) and were cultured until day 14. After day 14 and for 2 days, the cells were transferred to the third medium, which was also based on IMDM GlutaMAX medium supplemented with BSA (1%; MilliporeSigma), insulin (0.01 mg/mL; MilliporeSigma), transferrin human (0.2 mg/mL; MilliporeSigma), β-mercaptoethanol (91 μM), penicillin/streptomycin (1%), lipid mix 1 (1×; MilliporeSigma), ethanolamine (0.004%; MilliporeSigma), and hEPO (10 U/mL).

Counting and flow cytometric analysis of cells were performed on days 3, 5, 7, 10, and 14. During erythroid differentiation, cells were maintained at a concentration between 4 × 10^5^ and 4 × 10^6^ cells/mL. The incubation conditions were 37°C, 5% CO_2_, and 95% relative humidity.

Differentiation toward the erythroid lineage was analyzed by flow cytometry using a combination of antibodies, CD36-PE (BD Pharmingen), CD45-APCCy7 (BioLegend), CD71-PECy5 (BD Pharmingen), and CD235-PECy7, at 1 mL in 100 mL.

#### Clonogenic assays.

The number of hematopoietic progenitors in peripheral blood and BM in patients with DBA was evaluated according to the following protocol. Mononuclear hematopoietic cells and/or CD34^+^ cells were resuspended in X-vivo medium and seeded in semisolid methylcellulose medium (StemMACS HSC-CFU supplemented with 30% FCS, 1% BSA, 2 mM glutamine, 2-mercaptoethanol 0.1 nM, SCF 50 ng/mL, GM-CSF 20 ng/mL, G-CSF 20 ng/mL, IL-3 20 ng/mL, IL-6 20 ng/mL, EPO 3 U/mL; Miltenyi Biotec) at a concentration of 3 × 10^5^ nucleated cells/mL for peripheral blood and 5 × 10^4^ nucleated cells/mL for BM in 25 mm^2^ culture plates. After 14 days of incubation in hypoxia (37°C, 5% O_2_ and 5% CO_2_, 98% relative humidity), the colonies were counted using a phase contrast Nikon ELWD 0.3 inverted microscope. The results were expressed as the average of the total number of colonies per 10^5^ cells seeded.

#### Transplantation of human hematopoietic cells in immunodeficient mice.

Mice were housed and bred at the CIEMAT Animal Facility (registration number ES280790000183), where they were routinely screened for pathogens in accordance with the Spanish Society for the Laboratory Animal Science and the Federation of European Laboratory Animal Science Associations recommendations, and no pathogens were found. Mice were maintained under standard diet and food ad libitum. Mice were housed during the experimental protocols in micro-insulator individually ventilated cages type IIL with 25 air cage changes per hour. A maximum of 6 mice were housed in each cage. Room lighting was controlled with 13-hour light/11-hour dark cycles, and temperature and humidity were regulated at 20 ± 2°C and 55% ± 10%, respectively. HEPA air filters were present in all rooms.

Transplantation of HSCs was performed in NOD immunodeficient NSG and in NBSGW mice. Prior to engraftment, NSG mice were irradiated with a submyeloablative dose (1.5 Gy) and transplanted with 2 × 10^5^ purified CD34^+^ cells. In the case of the NBSGW strain, mice were irradiated with 1 Gy and transplanted with 3 × 10^5^ purified CD34^+^ cells and 1 × 10^6^ CD34^–^ cells irradiated with 30 Gy. We analyzed the hematopoietic graft in the NSG mice in peripheral blood and BM at days 30, 60, and 90 and in the case of the safety tests up to day 120 postinfusion by flow cytometry to assess the level of human hematopoietic graft. In the NBSGW strain, hematopoietic graft and lineage differentiation was evaluated at 30, 45, and 60 days. Cells were harvested by femoral BM aspiration at 4, 8, and 12 weeks after transplantation and labeled with the hCD45 APC-Cy7 antibody (eBioscience, Thermo Fisher Scientific). In 1 specific mouse, macrophage depletion to analyze the engraftment of erythroid lineage was performed by IV administration of clodronate liposomes that induce macrophage depletion in vivo. Macrophage depletion avoids the destruction of human erythropoiesis in the spleen of NSG, allowing us to see mature erythroid cells engrafted in this mouse model. One clodronate injection of 100 μL in 2 consecutive weeks at day 90 posttransplantation was performed. After 2 weeks the mouse was sacrificed and BM cells were harvested. Multilineage graft analyses were performed using CD45 APC-Cy7 (BioLegend), CD34 APC (BD Biosciences), CD33 PE (BD Biosciences), CD19 PE-Cy7, and CD3 FITC (BioLegend). Erythroid lineage analyses were performed using CD71.PE (Beckman Coulter) and CD235a-FITC (Beckman Coulter) (Supplemental Table 1) according to manufacturers’ instructions. Paired fluorochrome isotypes were used as controls. DAPI^+^ cells were excluded from the analysis. All flow cytometry analyses were performed on the Fortessa LSR and analyzed with FlowJo v7.6.5 software. To carry out the secondary transplantation, primary NSG recipients were anesthetized, and BM cells were obtained. Then human CD45^+^ cells were selected by cell separation by flow cytometry. Once purified, the cells were transplanted back into immunodeficient 1.5 Gy–irradiated NSG mice. The analyses were carried out at 30, 60, 90, and 120 days posttransplantation.

### IS analysis

The analysis of these integrations was carried out both in the cells expanded in vitro for 14 days, as well as in the cells grafted in primary and secondary NSG recipients of both experiments. The analyses were conducted by ProtaGene. The technique used by ProtaGene is based on shearing extension first tag selection ligation-mediated PCR, which amplifies and sequences unknown regions that flank the integrated vector DNA. DNA samples are fragmented by sonication into 500 bp fragments, then amplified by PCR using biotinylated primers specific for the *LTR* sequences of the integrated vector. After amplification, the biotinylated products are purified; the products are ligated into cassettes that include molecular labels (barcodes) and are subjected to a second step of PCR amplification with primers that allow sequencing using MiSeq technology (Illumina). For IS analysis, the 10 most frequent ISs were used.

### Statistics

Statistical analysis was performed with GraphPad Prism, version 7.0 for Windows. Previous statistical studies were carried out to check if the data followed a normal distribution using the statistical analysis of columns and applying the D’Agostino-Pearson normality test, Shapiro-Wilk normality test, and Kolmogórov-Smirnov normality test. Once the distribution in the different analyses had been studied, the most appropriate test was applied. These were 2-tailed Mann-Whitney statistical test, Student’s *t* test, multiple-comparison Kruskal-Wallis after normality test, and ANOVA test. Differences were considered significant when *P* < 0.05 or *P* ≤ 0.017 in the case of the Kruskal-Wallis test.

### Study approval

#### Animal procedures.

Procedures involving genetically modified organisms were conducted according to the proper European and Spanish regulations: Directive 2009/41/CE and Spanish Law 9/2003 and R.D. 178/2004. Procedures were approved by the Animal Experimentation Ethical Committee at CIEMAT according to all external and internal biosafety and bioethics guidelines and previously authorized by the Spanish Government (Code PROEX #070-15# Cell and Gene Therapy in rare diseases with chromosomal instability).

#### Human samples from HDs and DBA patients.

The human samples used in the results presented were peripheral blood and BM, both from HDs and from patients with DBA, as well as umbilical cord blood from HDs collected under written informed consent that was approved by the ethics committee of the corresponding hospital and project. The study meets the requirements established in current legislation (Royal Decree 1090/2015 and Decree 39/94 of the Comunidad de Madrid) and meets the standard ethical standards of the institution in this type of study and the ethical precepts formulated in SAS Order 3470/2009 and the Declaration of Helsinki of the World Medical Association.

### Data availability

Values for all data points found in graphs are in the Supporting Data Values file.

## Author contributions

YG, MP, RSD, DLJL, JB, and SN conceived and designed the experiments. YG, MP, CZ, LU, RSD, JP, OA, MV, and SN performed experiments and collected and assembled data. PR, EG, AP, DLJL, and RSD provided reagents, materials, complementary data, analysis tools, and ideas. LDC, MS, AC, ARL, JCS, JS, BS, CMN, CB, and TL provided patient and healthy donor samples, clinical data, and constructive discussion and ideas. YG, CZ, DLJL, JB, and SN wrote, edited, and revised the manuscript and produced the final figures.

## Supplementary Material

Supplemental data

Supporting data values

## Figures and Tables

**Table 1:**
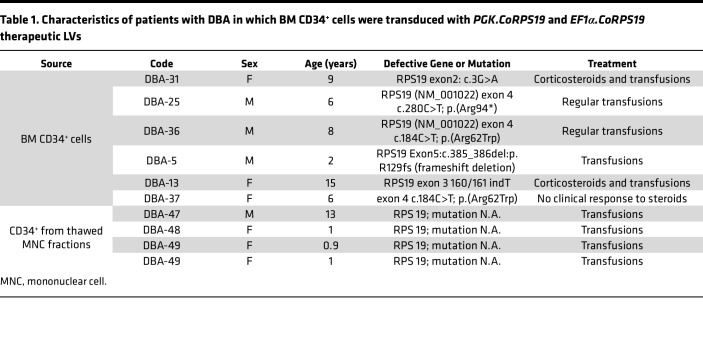
Characteristics of patients with DBA in which BM CD34^+^ cells were transduced with *PGK.CoRPS19* and *EF1α.CoRPS19* therapeutic LVs

**Table 2:**
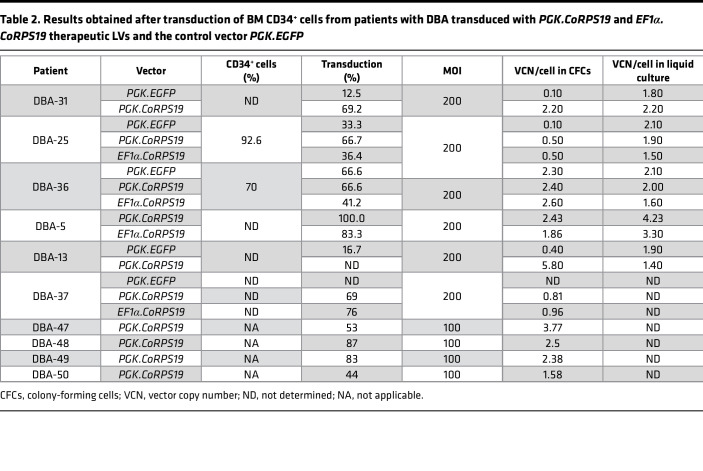
Results obtained after transduction of BM CD34^+^ cells from patients with DBA transduced with *PGK.CoRPS19* and *EF1α.CoRPS19* therapeutic LVs and the control vector *PGK.EGFP*

## Version 1 05/22/2024

Electronic publication
