# Supplementary material for: Lentivirus-mediated gene therapy corrects ribosomal biogenesis and shows promise for Diamond Blackfan anemia
Source: JCI Insight. 2024 May 22;9(10):e171650. doi: 10.1172/jci.insight.171650 (PMC11141922; doi:10.1172/jci.insight.171650)
Supplement: Supplemental data [file jciinsight-9-171650-s026.pdf]

a)

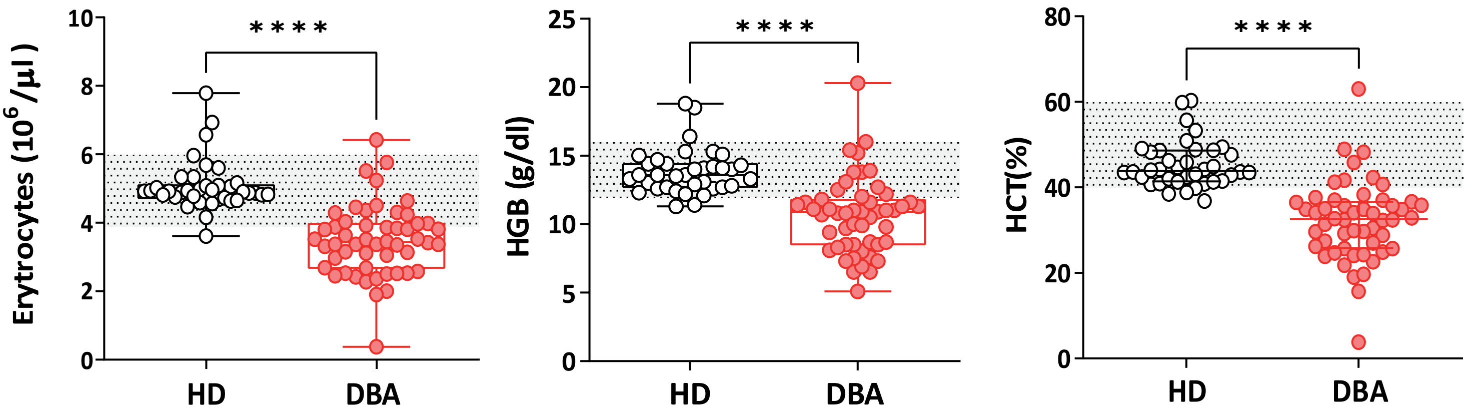

Erytrocytes (10^6/µl)
HGB (g/dl)
HCT(%)
****
HD
DBA

b)

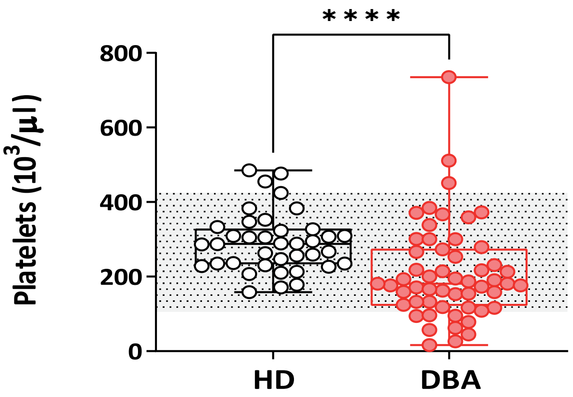

Platelets (10^3/µl)
****
HD
DBA

c)

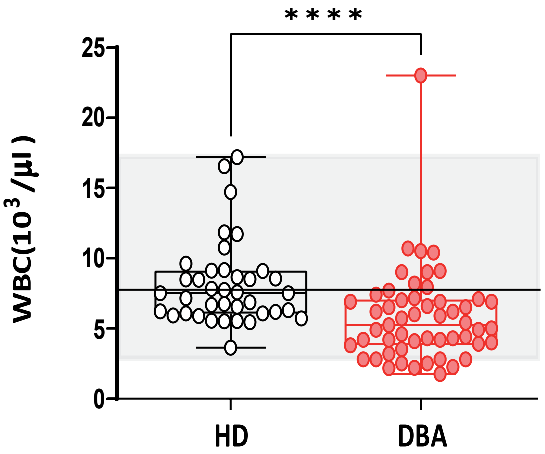

WBC(10^3/µl)
****
HD
DBA

Supplemetary Figure 2

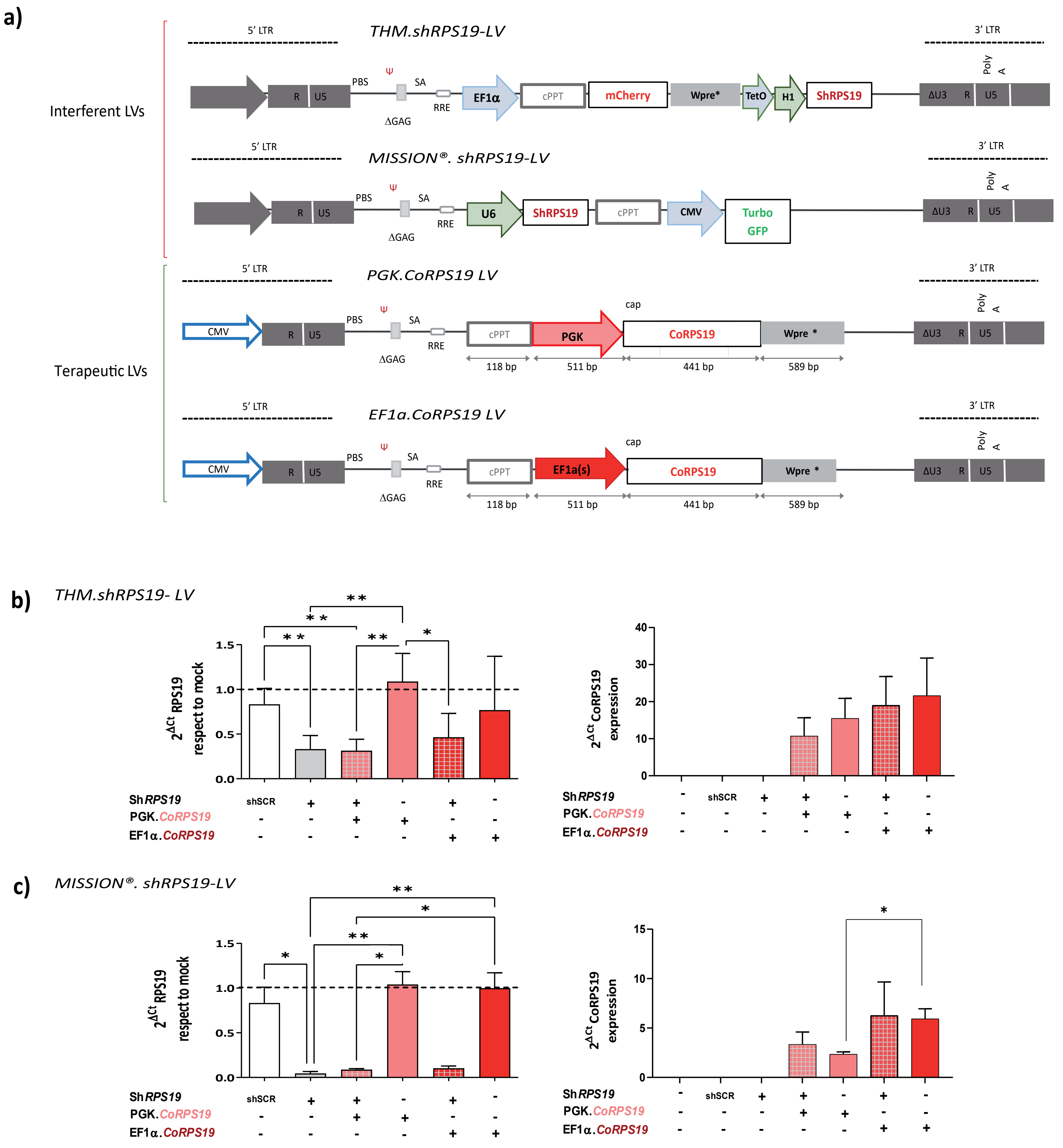

Supplemetary Figure 3

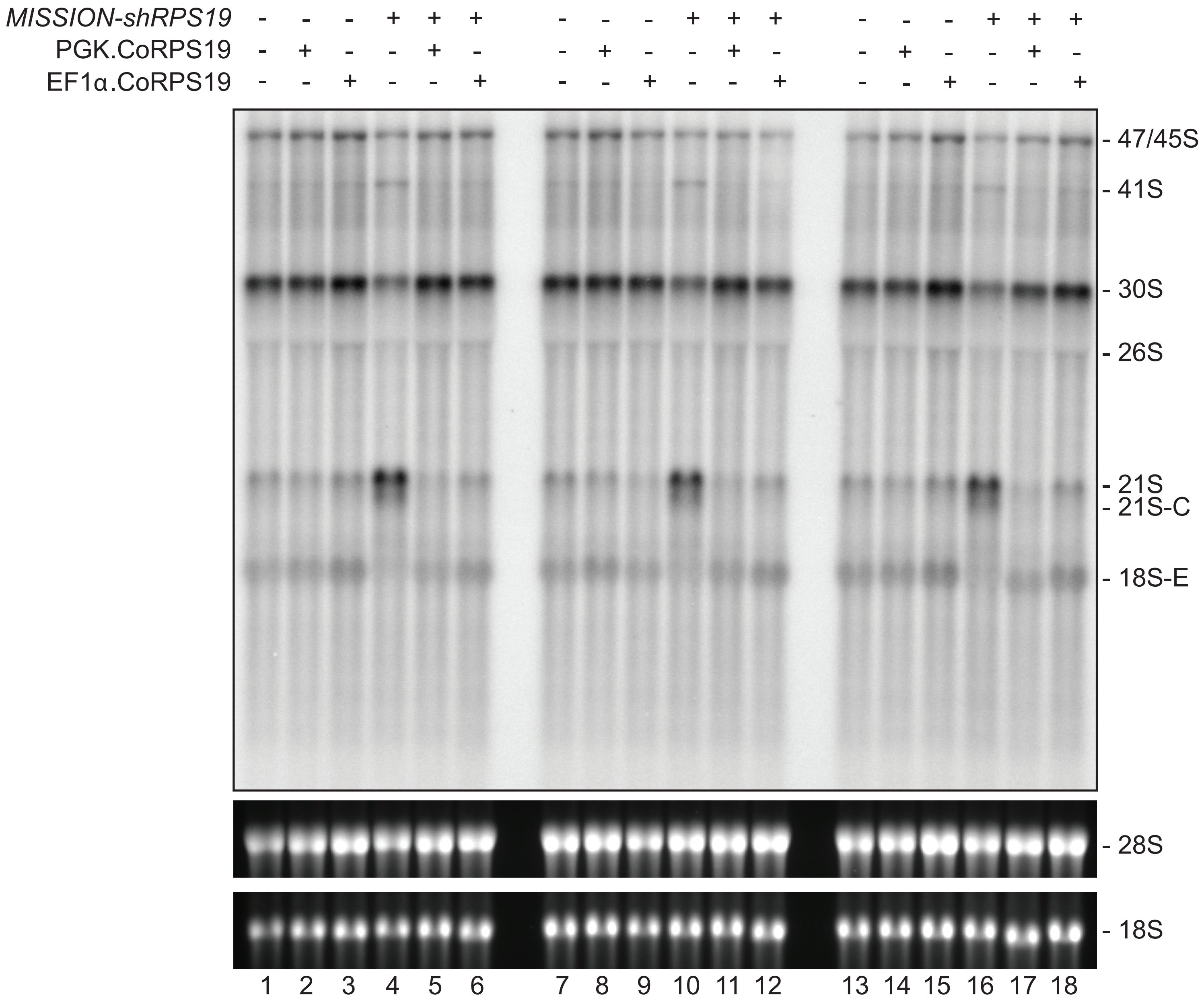

Supplemetary Figure 4

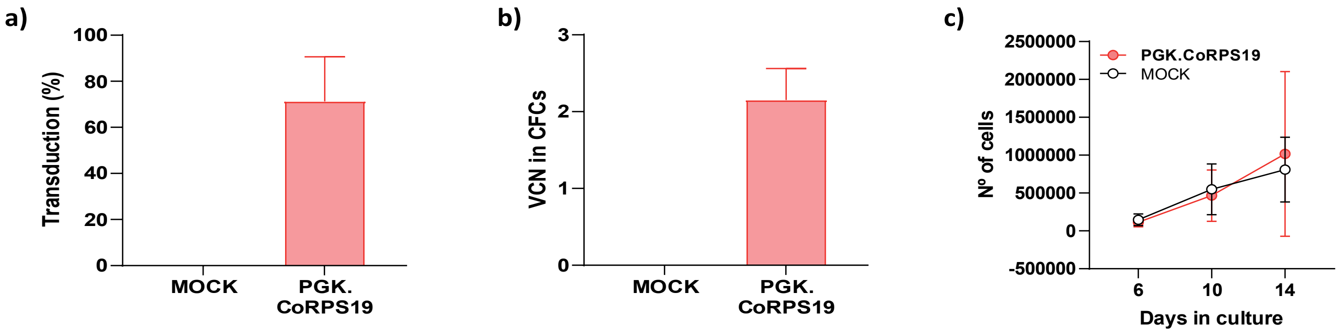

a)

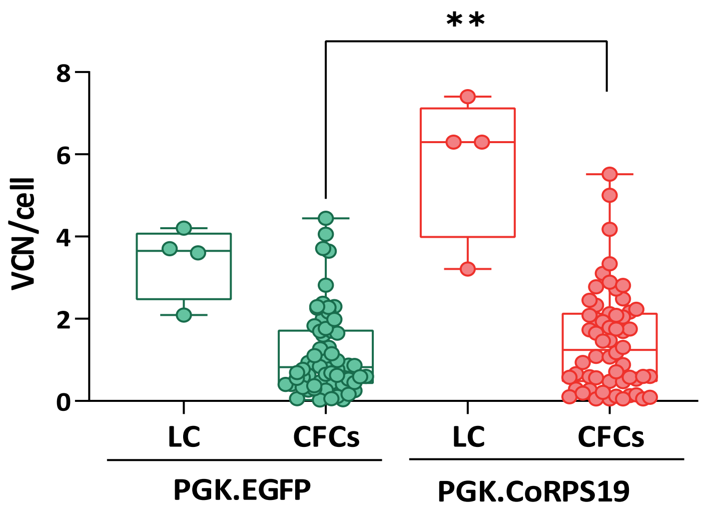
**
8
6
4
2
0
VCN/cell
LC
CFCs
LC
CFCs
PGK.EGFP
PGK.CoRPS19

b)

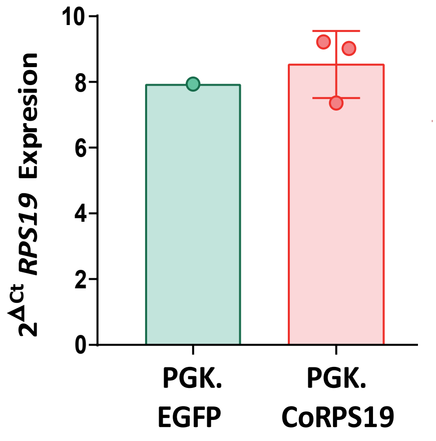
10
8
6
4
2
0
2ΔCt RPS19 Expresion
PGK.
EGFP
PGK.
CoRPS19

c)

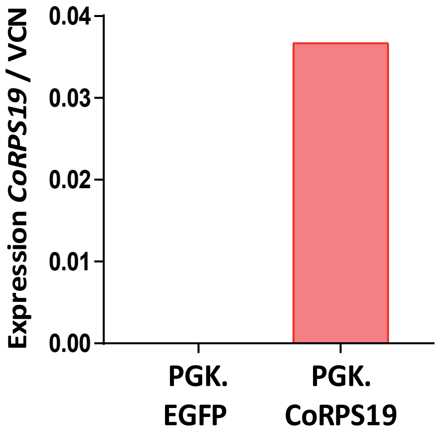
0.04
0.03
0.02
0.01
0.00
Expression CoRPS19 / VCN
PGK.
EGFP
PGK.
CoRPS19

Supplemetary Figure 6

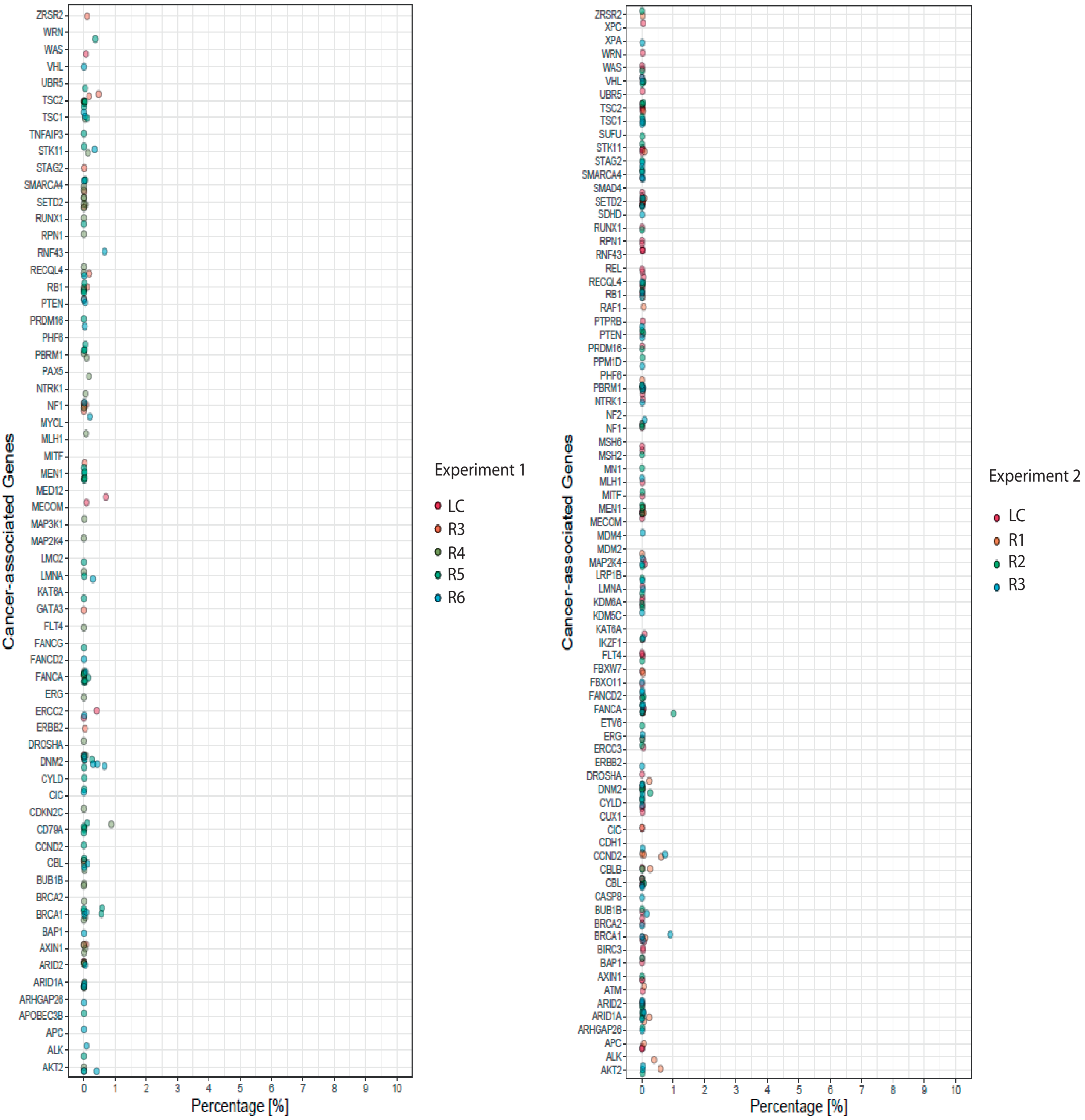

Supplementary Figure legends

Figure S1. Determination of several hematological parameters in peripheral blood of DBA patients and healthy donors

a) Number of erythrocytes (10^6/µl), hemoglobin concentration (g/dl) and hematocrit (%).

b) Number of platelets/µl in the peripheral blood of DBA patients as compared to HDs.

c) White blood cells (WBCs) per µl in the peripheral blood of DBA patients compared to HDs.

In all panels, the gray areas mark the range of mean clinical values for the respective hematologic parameters. The degree of significance was determined with the Mann Whitney test (p-value *<0.05; **< 0.01; *** <0.001; **** < 0.0001). The graphs show the median and interquartile range along with the 90th and 10th percentiles. HD: healthy donors; DBA: DBA patients.

Figure S2. Schematic diagram of the shRNA interference vectors and therapeutic vectors generated

a) Schematics of the lentiviral interference vectors used in the study (vector LV-THM.sh7FANCA, described by Dr. Flygare, and vector MISSION® pLKO.1-pure vector TurboGFP™ shRPS19, purchased from Merk) and of the two developed clinically applicable therapeutic vectors PGK.CoRPS19 LV and EF1α.CoRPS19 LV.

5' LTR, 5' long terminal repeat; PBS, primer binding site; Ψ, psi packaging signal; ΔGAG, truncated Gag sequence; SA, splicing acceptor; RRE, Rev responsive element (RRE); EF1α, elongation factor 1α; mCherry, monomeric red fluorescent proteins (mRFPs); TurboGFP, bright dimeric green fluorescent protein derived from CopGFP; TetO,

tetracycline inducible promoter; Expression H1, H1 RNA polymerase III (Pol III) promoter widely used for the expression of small noncoding RNAs; ShRPS19, short hairpin RNA targeting endogenous RPS19; U6, RNA polymerase III (PolIII) promoter widely used for the expression of small noncoding RNAs; CMV, cytomegalovirus promoter; cPPT, DNA flap central polypurine tract; PGK, phosphoglycerate kinase promoter; EF1α(s), elongation factor 1α in its short version; CoRPS19, codon optimized nucleotide sequence encoding the RPS19 protein; Wpre*, mutated optimized woodchuck hepatitis virus posttranscriptional regulatory element; 3' LTR, 3' long terminal repeat; PolyA, polyadenylation signal sequence.

b) Left: reduction of endogenous RPS19 expression by means of the vector LV-THM.shRPS19 (ShRPS19-LV); Right: subsequent detection of the CoRPS19 sequence present in therapeutic vectors PGK.CoRPS19-LV and EF1α.CoRPS19 LV.

c) Left: reduction of endogenous expression of RPS19 by means of the vector LV-MISSION® pLKO.1-pure TurboGFP™ shRPS19 (ShRPS19 LV). Right: subsequent detection of the CoRPS19 sequence present in the therapeutic vectors PGK.CoRPS19 LV and EF1α(s).CoRPS19 LV.

Figure S3. The therapeutic vectors *PGK.CoRPS19 LV* and *EF1α.CoRPS19 LV* are functional

The data shows the uncropped northern blot panels of Figure 2b revealing the consistent (triplicate analysis) restoration at the level of 41S, 21S/21S-C, and 18S-E pre-rRNA levels.

Figure S4. Transduction fitness in CD34$^+$ from DBA patients coming from thawed MNC fractions and transplanted in *NBSGW* mice.

a) Transduction efficacy in CD34+ cells purified from frozen MNCs of DBA-patients with *PGK.CoRPS19 LV* (n=4) o in not transduced condition (Mock=4) The data are presented as means with standard deviation.

b) Number of integrated copies of proviral genome per cell (vector copy number, VCN) in colony-forming cells (CFCs) at 14 days of culture.

c) Expansion in erythroid medium, of hematopoietic progenitors from DBA-patient BM after successful tranduction with the therapeutic vector *PGK.CoRPS19 LV*. Cell number was measured before transduction at day 6 and after 710 and 14 days in liquid culture.

Figure S5. Complementary safety analyses performed after transfection of BM CD34+ cells from RPS19-deficient patients with the therapeutic vector PGK.CoRPS19 LV

a) Determination of the vector copy number (VCN) per HD CB CD34+ cell in CFCs.

b) Expression of endogenous RPS19.

c) Expression of CoRPS19 normalized for the VCN per cell. Statistical significance was determined with the Mann Whitney test (p-value; * <0.05;_** <0.01; *** <0.001; **** <0.000105).

Figure S6. Relative contribution of ISs within a 100-kb window around the TSS of cancer-associated genes in experiments 1 and 2

Each dot represents a gene harboring an IS within 100 kb of the TSS, with the relative contribution of the IS to the total read counts shown on the x-axis. The y-axis lists all selected cancer-associated genes harboring an IS within 100 kb of the TSS. For each

cancer-associated gene, data from different samples are displayed in different colors as indicated. TSS, transcription start site.

Table S1. Panel of antibodies used for the immunophenotype of patients with DBA and healthy donors.

Panel	Epitope	Fluorochrome	Clone	Supplier	Catalogue	V_{final} (μL)	V_{ab}/V_{final}
1: Erythroid progenitos	CD71	PE	YDJ.1.2.2	Beckman Coulter	IM2001U	50	2.5
	CD235a	FITC	11E4B-7-6	Beckman Coulter	B49206		5
2: Megakaryocytes and platelets	CD41a	PE	B61391	BD Pharimigen	555472	50	5
	CD42b	FITC	4336556	Beckman Coulter	A07781		5
3: Hematopoietic progenitors	CD34	PE	8G12	BD Pharimigen	345802	100	4
	CD38	FITC	T16	Immunotech	IM0775		4
	CD45	APC	2D1	Biolegend	368512		5

Table S2. Panel of antibodies used for the determination of hematopoietic progenitors in bone marrow.

	TUBE #	CD15S BV421 (h203)	CD49f BV510 (h207)	HECA452 BV605 (h204)	CD135 BV711 (h205)	CD10 FITC (h18)	PI (20µg/ml)	CD38 PECy5.5 (h32)	CD7 PE (h14)	CD34 PECY7 (h45)	CD90 APC (h164)	CD45RA APCe fluor780 (h194)
COMPENSATION CONTROLS (200,000 cells negative fraction)	1: CD15S	2 µl	----	----	----	----	----	----	----	----	----	----
	2: CD49f	----	2 µl	----	----	----	----	----	----	----	----	----
	3: HECA452	----	----	2 µl	----	----	----	----	----	----	----	----
	4: CD135	----	----	----	2 µl	----	----	----	----	----	----	----
	5: CD10	----	----	----	----	2 µl	----	----	----	----	----	----
	6: PI	----	----	----	----	----	PI	----	----	----	----	----
	7: CD38	----	----	----	----	----	----	1.5 µl	----	----	----	----
	8: CD7	----	----	----	----	----	----	----	2 µl	----	----	----
	9: CD34	----	----	----	----	----	----	----	----	1.5 µl	----	----
	10: CD90	----	----	----	----	----	----	----	----	----	1.5 µl	----
	11: CD45RA	----	----	----	----	----	----	----	----	----	----	1.5 µl
FMOs (20,000 CD34^{+} cells or 200,000 cells negative fraction or 200,000 total BM)	12: CD15S	----	2 µl	2 µl	2 µl	2 µl	PI	1.5 µl	2 µl	1.5 µl	1.5 µl	1.5 µl
	13: CD49f	2 µl	----	2 µl	2 µl	2 µl	PI	1.5 µl	2 µl	1.5 µl	1.5 µl	1.5 µl
	14: HECA452	2 µl	2 µl	----	2 µl	2 µl	PI	1.5 µl	2 µl	1.5 µl	1.5 µl	1.5 µl
	15: CD135	2 µl	2 µl	2 µl	----	2 µl	PI	1.5 µl	2 µl	1.5 µl	1.5 µl	1.5 µl
	16: CD10	2 µl	2 µl	2 µl	2 µl	----	PI	1.5 µl	2 µl	1.5 µl	1.5 µl	1.5 µl

	**17: PI**	**2 µl**	**2 µl**	**2 µl**	**2 µl**	**2 µl**	**----**	**1.5 µl**	**2 µl**	**1.5 µl**	**1.5 µl**	**1.5 µl**
	**18: CD38**	**2 µl**	**2 µl**	**2 µl**	**2 µl**	**2 µl**	**PI**	**----**	**2 µl**	**1.5 µl**	**1.5 µl**	**1.5 µl**
	**19: CD7**	**2 µl**	**2 µl**	**2 µl**	**2 µl**	**2 µl**	**PI**	**1.5 µl**	**----**	**1.5 µl**	**1.5 µl**	**1.5 µl**
	**20: CD34**	**2 µl**	**2 µl**	**2 µl**	**2 µl**	**2 µl**	**PI**	**1.5 µl**	**2 µl**	**----**	**1.5 µl**	**1.5 µl**
	**21: CD90**	**2 µl**	**2 µl**	**2 µl**	**2 µl**	**2 µl**	**PI**	**1.5 µl**	**2 µl**	**1.5 µl**	**----**	**1.5 µl**
	**22: CD45RA**	**2 µl**	**2 µl**	**2 µl**	**2 µl**	**2 µl**	**PI**	**1.5 µl**	**2 µl**	**1.5 µl**	**1.5 µl**	**----**
**PURIFIED SAMPLE (50,000 $CD34^+$ cells)**	**23A: Sample**	**2 µl**	**2 µl**	**2 µl**	**2 µl**	**2 µl**	**PI**	**1.5 µl**	**2 µl**	**1.5 µl**	**1.5 µl**	**1.5 µl**
**TOTAL SAMPLE (BM, mPB) ($\geq 2*10^6$ total cells)**	**23B: Sample**	**3 µl**	**3 µl**	**3 µl**	**3 µl**	**3 µl**	**PI**	**2.5 µl**	**3 µl**	**2.5 µl**	**2.5 µl**	**2.5 µl**
